# Pioglitazone Modulates the Vascular Contractility in Hypertension by Interference with ET-1 Pathway

**DOI:** 10.1038/s41598-019-52839-6

**Published:** 2019-11-11

**Authors:** Roberto Palacios-Ramírez, Raquel Hernanz, Angela Martín, José V. Pérez-Girón, María T. Barrús, Zoe González-Carnicero, Andrea Aguado, Frederic Jaisser, Ana M. Briones, Mercedes Salaices, María J. Alonso

**Affiliations:** 1 Depto. de Ciencias Básicas de la Salud, Facultad de Ciencias de la Salud, Universidad Rey Juan Carlos, Alcorcón, Spain; 2 CIBER de Enfermedades Cardiovasculares, Madrid, Spain; 3 Depto. de Farmacología, Facultad de Medicina, Universidad Autónoma de Madrid, Instituto de Investigación Hospital La Paz (IdiPaz), Madrid, Spain; 4 Institut National de la Santé et de la Recherche Médicale Inserm U1138, Cordeliers Institute, Paris VI-University, Paris, France

**Keywords:** Cardiovascular diseases, Hypertension

## Abstract

Endothelin-1 (ET-1) is an important modulator of the vascular tone and a proinflammatory molecule that contributes to the vascular damage observed in hypertension. Peroxisome-proliferator activated receptors-γ (PPARγ) agonists show cardioprotective properties by decreasing inflammatory molecules such as COX-2 and reactive oxygen species (ROS), among others. We investigated the possible modulatory effect of PPARγ activation on the vascular effects of ET-1 in hypertension. In spontaneously hypertensive rats (SHR), but not in normotensive rats, ET-1 enhanced phenylephrine-induced contraction through ET_A_ by a mechanism dependent on activation of TP receptors by COX-2-derived prostacyclin and reduction in NO bioavailability due to enhanced ROS production. In SHR, the PPARγ agonist pioglitazone (2.5 mg/Kg·day, 28 days) reduced the increased ET_A_ levels and increased those of ET_B_. After pioglitazone treatment of SHR, ET-1 through ET_B_ decreased ROS levels that resulted in increased NO bioavailability and diminished phenylephrine contraction. In vascular smooth muscle cells from SHR, ET-1 increased ROS production through AP-1 and NFκB activation, leading to enhanced COX-2 expression. These effects were blocked by pioglitazone. In summary, in hypertension, pioglitazone shifts the vascular ET_A_/ET_B_ ratio, reduces ROS/COX-2 activation and increases NO availability; these changes explain the effect of ET-1 decreasing phenylephrine-induced contraction.

## Introduction

Peroxisome-proliferator activated receptors (PPARs) are ligand-activated transcription factors that belong to the nuclear hormone receptor superfamily and regulate the expression of genes involved in glucose and lipids metabolism^1^. PPARγ is expressed in all vascular cells, including vascular smooth muscle cells (VSMC)^2^. PPARγ activation reduces reactive oxygen species (ROS) production and inflammatory molecules such as cyclooxygenase-2 (COX-2) by interfering with mitogen-activated protein kinases (MAPK) and/or proinflammatory transcription factors^3–6^, explaining the cardioprotective effect of PPARγ agonists^7^. In this sense, PPARγ ligands antagonize the vascular damage and functional alterations observed in inflammatory pathologies such as hypertension^8–10^.

Endothelin-1 (ET-1), the most important isoform of the endothelin family, is mainly produced by endothelial cells, although other vascular cells, including VSMC, release ET-1 in response to different stimuli^11,12^. ET-1 interacts with two different G-protein-coupled receptors, ET-1 receptor A (ET_A_) and ET-1 receptor B (ET_B_). In the vasculature, ET_A_ receptors are located in the smooth muscle cells and cause sustained vasoconstriction; ET_B_ predominate in endothelial cells and mediate vasodilatation by release of endothelium-derived relaxing factors (nitric oxide -NO-, prostacyclin-PGI_2_- and/or endothelium-derived hyperpolarizing factor), although a small population of this subtype is present in VSMC causing vasoconstriction in certain vascular beds^13^. In addition to the mentioned vasoconstrictor effect, ET-1 stimulates the renin-angiotensin-aldosterone and sympathetic nervous systems and possesses cardiac positive inotropic and chronotropic effects; then, ET-1 has a pivotal role at cardiovascular level^14^. It is well known that ET-1 contributes to the pathophysiological alterations observed in different inflammatory disorders, such as hypertension^15–17^. Thus, endogenous ET-1 seems to mediate some of the deleterious cardiovascular effects attributed to angiotensin II, such as the renal effects, the cardiac fibrosis or hypertrophy^18–20^. Besides its vasoconstrictor effect, subthreshold concentrations of ET-1 increase the contractility to other vasoactive agents^21–23^. Furthermore, ET-1 increases oxidative stress and proinflammatory enzymes such as COX-2^3,5,24–27^ that are involved in the hypertension-associated vascular damage^28–30^.

PPARγ activation reduces ET-1 levels^31,32^; in this sense, we have described that, by downregulating ET-1 transcription, pioglitazone inhibits angiotensin II-associated vascular COX-2 expression in hypertension^5^. However, the effect of PPARγ activation on the vascular actions of ET-1 is less known. We hypothesize that glitazones contribute to reduce the hypertension-associated vascular damage by interfering with ET-1-induced vasoactive and proinflammatory effects. For this, in vessels or VSMC from hypertensive rats we have analysed: (1) the effect of a subthreshold concentration of ET-1 on phenylephrine responses as well as the role of NO and COX-2-derived prostanoids in such effect; (2) the mechanisms involved in the ET-1-induced COX-2 expression; (3) the interference induced by the PPARγ agonist pioglitazone of these ET-1 effects.

## Results

### ET-1 enhances phenylephrine contraction in resistance arteries from hypertensive rats via ET_A_

**Figure 1:**
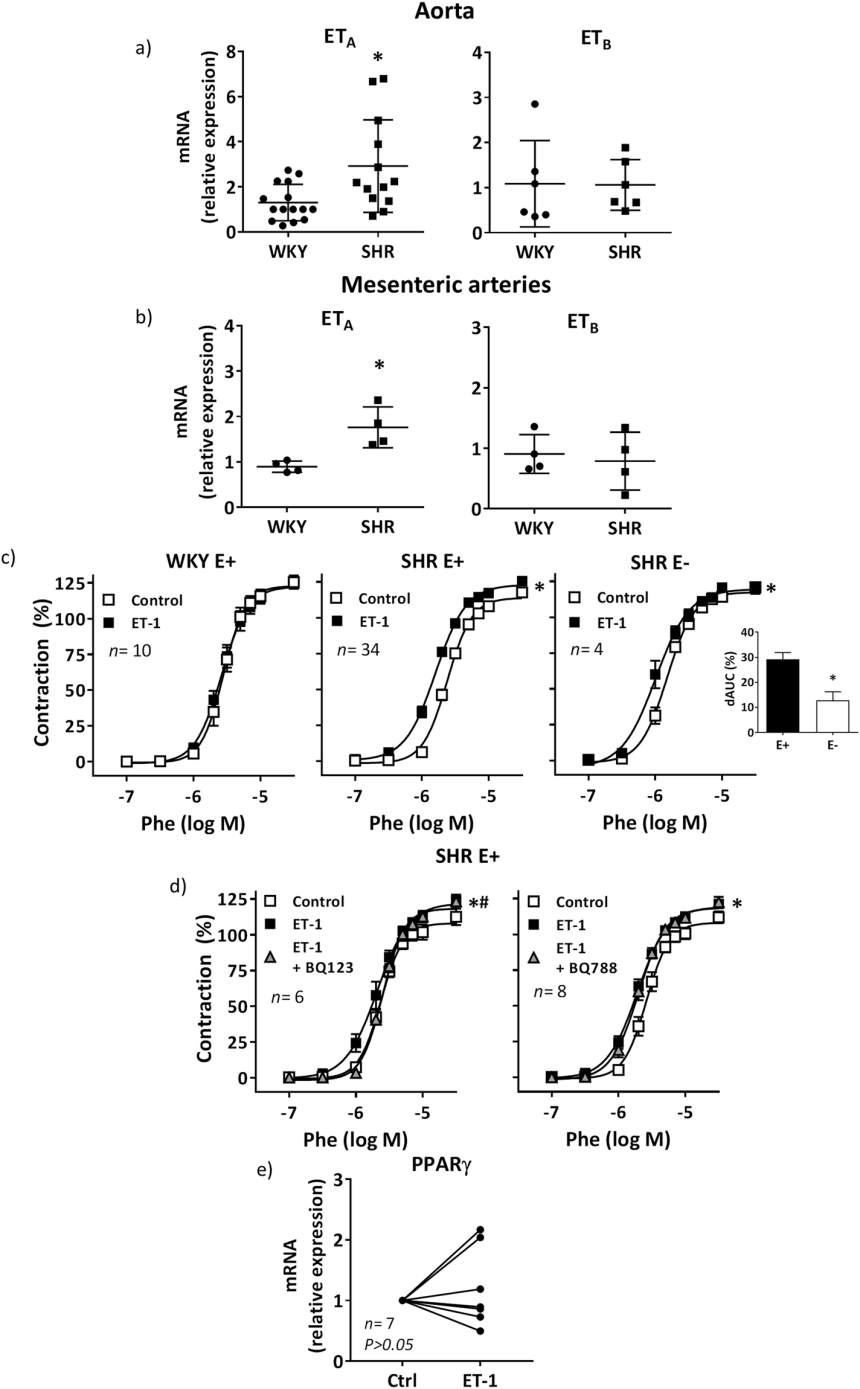
Vascular ET-1 receptors expression and effect of ET-1 on phenylephrine contraction in MRA. ET_A_ and ET_B_ mRNA levels in aortic (**a**) and mesenteric (**b**) segments from WKY and SHR; *P < 0.05 by Student's t test. (**c**) Effect of ET-1 (1 nM, 90 min) on the concentration-response curve to phenylephrine (Phe) in endothelium-intact (E+) and denuded (E-) segments from WKY and SHR; *P < 0.05 by two-way ANOVA with repeated measures followed by Sidak post hoc test. Differences of the area under the concentration-response curve (dAUC) to Phe in E+ and E- segments from SHR are also shown; *P < 0.05 by Student's t test. (**d**) Effect of BQ123 (1 µM) and BQ788 (1 µM) on the response to Phe in E+ segments from SHR treated with ET-1; *P < 0.05 vs control; ^#^*P* < 0.05 vs ET-1 by two-way ANOVA with repeated measures followed by Tukey post hoc test. (**e**) Effect of ET-1 on the vascular expression of PPARγ; statistical analysis by Student's t test. *n* denotes number of experiments.

In a previous report, we found greater aortic ET-1 mRNA levels in spontaneously hypertensive rats (SHR) compared to Wistar Kyoto (WKY) rats^5^. Here, we also found greater ET_A_ mRNA levels in both aortic (Fig. 1a) and mesenteric segments (Fig. 1b) from SHR than WKY, while those of ET_B_ were similar in both strains (Fig. 1a,b). In order to analyse whether this different receptor expression pattern has functional consequences, reactivity experiments were performed. ET-1, at a concentration (1 nM) which does not induce any vasoactive effect, enhanced the phenylephrine-induced contraction in mesenteric resistance arteries (MRA) from SHR (Emax: Control, 113.6 ± 2.2%; ET-1, 122.8 ± 2.3%, *P* < 0.05; pD_2_: Control, 5.62 ± 0.02; ET-1, 5.82 ± 0.02; *P* < 0.05), without affecting the contraction in WKY arteries (Fig. 1c). The effect of ET-1 on phenylephrine responses was diminished by endothelium removal in arteries from SHR, as shown by the lack of effect of ET-1 in Emax (Control, 117.6 ± 1.1%; ET-1, 119.6 ± 2.9%, *P*>0.05) and by the decreased dAUC values (Fig. 1c). The ET_A_ antagonist BQ123 (1 µM) abolished the effect of ET-1 in intact SHR segments; however, no effect of BQ788 (1 µM), an ET_B_ antagonist, was observed (Fig. 1d). On the other hand, ET-1 did not affect the vascular mRNA levels of PPARγ (Fig. 1e).

### Reduced NO bioavailability, likely by increase of oxidative stress, contributes to the ET-1-induced enhancement of phenylephrine contraction in MRA from SHR

**Figure 2:**
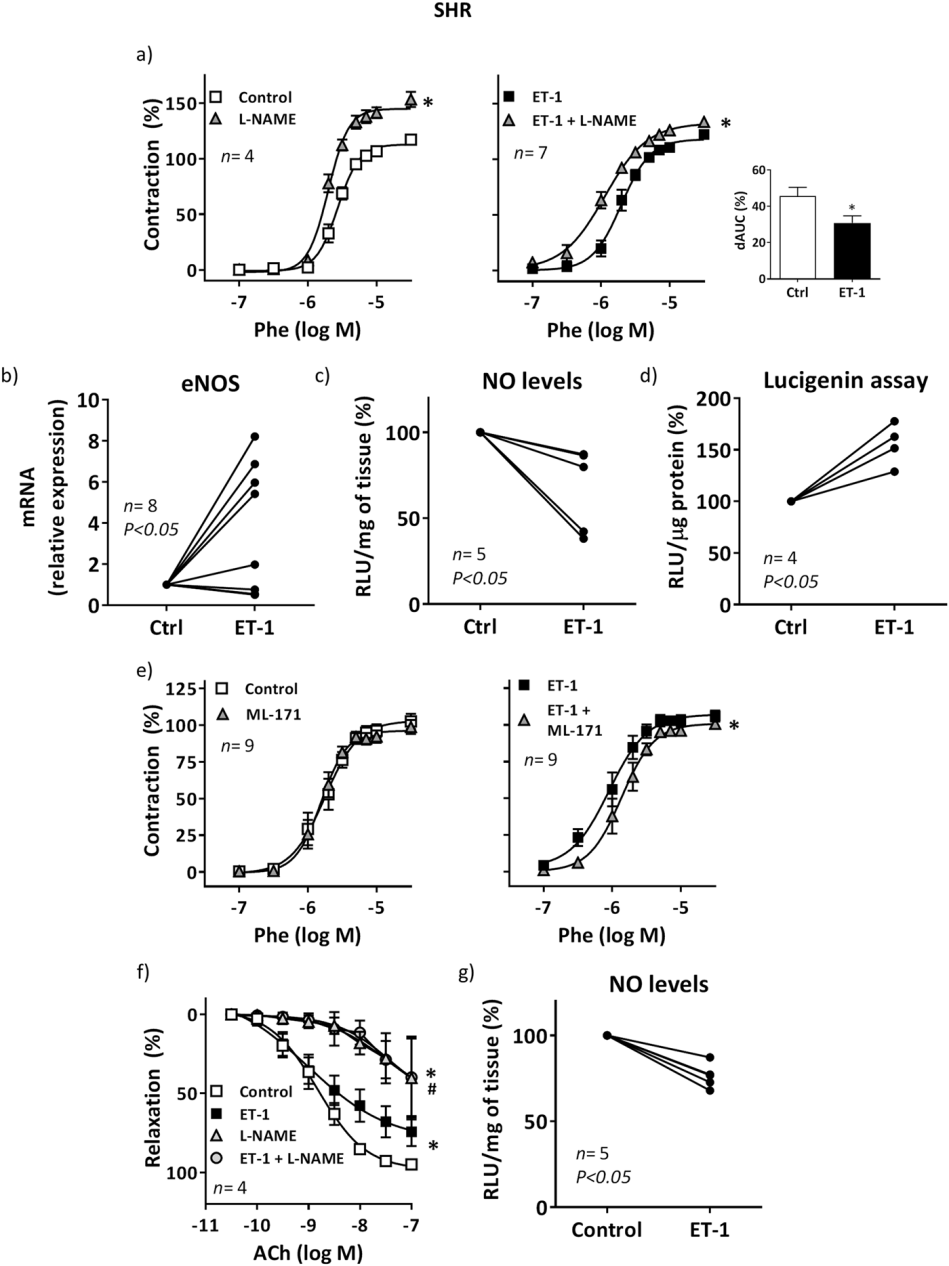
Role of nitric oxide and oxidative stress on ET-1-induced potentiation of phenylephrine response in MRA from hypertensive rats. (**a**) Effect of 0.1 mM L-NAME on the concentration-response curve to phenylephrine (Phe) in control and ET-1-incubated (1 nM, 90 min) segments from SHR; *P < 0.05 by two-way ANOVA with repeated measures followed by Sidak post hoc test. Differences of the AUC (dAUC) to Phe in the presence and the absence of L-NAME are also shown; *P < 0.05 by Student's t test. (**b**) Effect of ET-1 on eNOS mRNA levels in vascular segments; statistical analysis by Student's t test. (**c**) Quantification of nitric oxide (NO) release and (**d**) NADPH oxidase activity in control and ET-1-incubated segments from SHR; statistical analysis by Student’s t test. (**e**) Effect of 0.5 µM ML-171 on the concentration-response curve to Phe in control and ET-1-incubated segments from SHR; *P < 0.05 by two-way ANOVA with repeated measures followed by Sidak post-test. (**f**) Effect of ET-1 and L-NAME on the concentration-response curve to acetylcholine (ACh) in segments from SHR; *P < 0.05 vs Control; ^#^*P* < 0.05 vs ET-1 by two-way ANOVA with repeated measures followed by Sidak post hoc test. (**g**) Quantification of ACh-induced NO release in control and ET-1-incubated segments from SHR; statistical analysis by Student's t test. *n* denotes number of experiments.

The incubation with the NOS inhibitor N-nitro-L-arginine methyl ester (L-NAME, 0.1 mM) leftward shifted the phenylephrine contraction in MRAs from SHR, being this effect reduced in ET-1-treated arteries (Fig. 2a), suggesting that ET-1 reduces the negative modulation induced by NO on phenylephrine contraction; in agreement, and despite the increase of eNOS expression induced by ET-1 (Fig. 2b), the basal NO levels were lower in ET-1-incubated segments (Fig. 2c). It is well accepted that ET-1 increases oxidative stress at vascular level^24,26,27^. Accordingly, ET-1 augmented the vascular NADPH oxidase (NOX) activity (Fig. 2d) and the NOX inhibitor ML-171 (0.5 µM) reduced the phenylephrine-induced contraction only in ET-1-treated arteries (Fig. 2e). These results suggest that oxidative stress, by reducing NO bioavailability, would contribute to enhance the phenylephrine-induced response after ET-1 treatment. In addition, ET-1 impaired the vasodilator response induced by ACh (Fig. 2f). After L-NAME incubation ACh-induced relaxation was reduced to a similar level in both ET-1 untreated and treated segments (Fig. 2f), suggesting that ET-1 impaired the NO contribution to endothelium-dependent vasodilator responses. In agreement, ACh-induced NO levels were lower after ET-1 incubation than in control segments (Fig. 2g).

### COX-2 derivatives acting on TP receptors are involved in ET-1-induced potentiation of phenylephrine contraction in MRA from SHR

**Figure 3:**
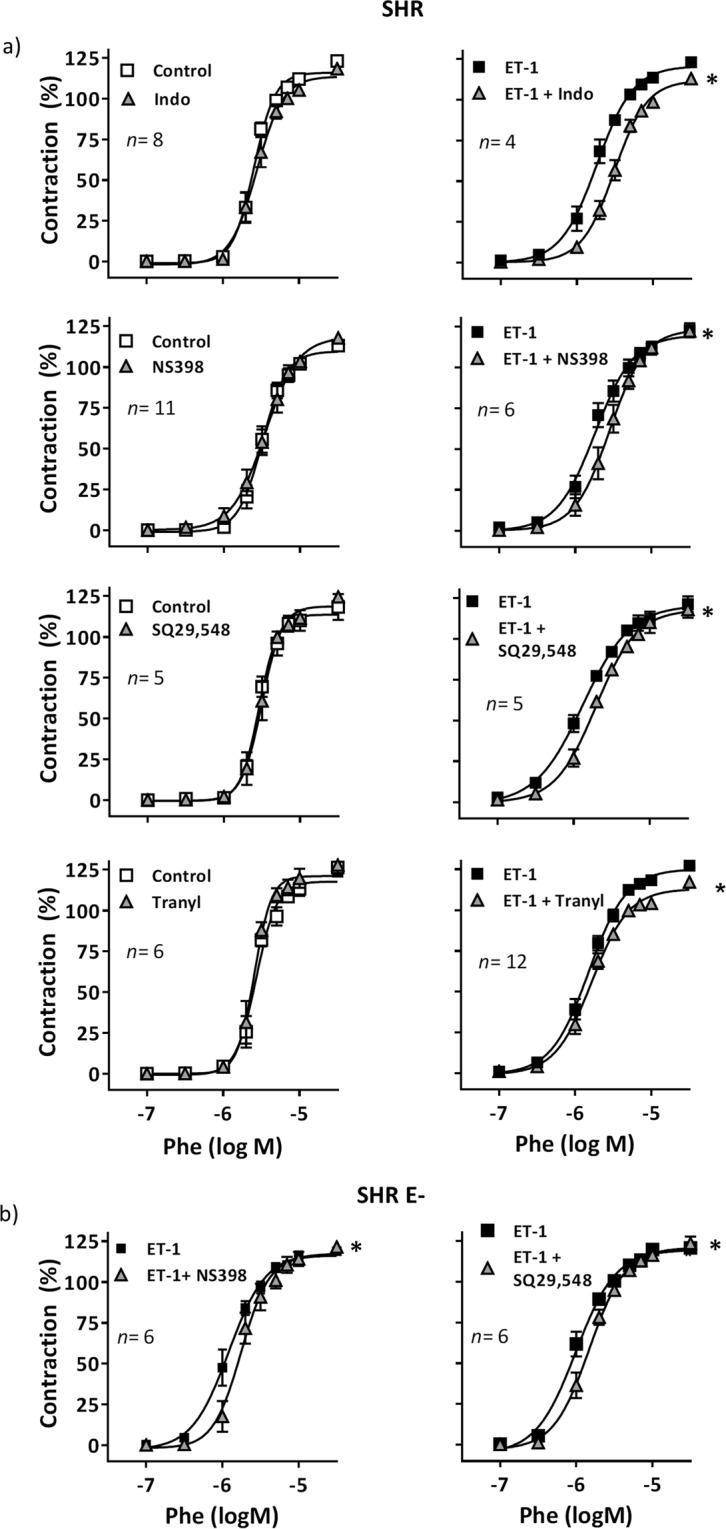
Role of cyclooxygenase-derived prostanoids on ET-1-induced potentiation of phenylephrine response in MRA from hypertensive rats. (**a**) Effect of 10 µM indomethacin (Indo), 1 µM NS398, 1 µM SQ29,548 and 10 µM tranylcypromine (Tranyl) on the concentration-response curve to phenylephrine (Phe) in intact control and ET-1-incubated (1 nM, 90 min) segments from SHR. (**b**) Effect of NS398 and SQ29,548 on the concentration-response curve to Phe in endothelium-denuded (E-) segments from SHR incubated with ET-1. *P < 0.05 by two-way ANOVA with repeated measures followed by Sidak post-test. *n* denotes number of experiments.

We have previously described that ET-1 increases COX-2 expression in VSMCs from SHR^5^. Then, we analysed the contribution of COX-derived prostanoids to the effect of ET-1 on phenylephrine responses in SHR arteries. Both the non-selective COX inhibitor indomethacin (10 µM) and the selective COX-2 inhibitor NS398 (1 µM), reduced phenylephrine contraction only in ET-1-treated segments (Fig. 3a), suggesting that COX-2-derived vasoconstrictor prostanoids contribute to the increase of phenylephrine response induced by ET-1 in hypertensive animals. The EP_1–3_ receptor antagonist SC19220 (10 µM) did not affect phenylephrine response neither in control nor in ET-1-incubated segments (data not shown); however, the TxA_2_ receptor (TP) antagonist SQ29,548 (1 µM) reduced phenylephrine contraction only in ET-1-incubated MRA (Fig. 3a). Notably, the TxA_2_ synthase inhibitor furegrelate (1 µM) did not affect phenylephrine-induced contraction in either control or ET-1-treated segments (data not shown), apparently excluding TXA_2_ as the vasoconstrictor prostanoid responsible for ET-1-induced hypercontractility. It is well known that PGI_2_ is the main COX-derived vasodilator prostanoid; however, in some conditions such as hypertension or aging, prostacyclin can induce vasoconstriction via TP receptors^10,33^. We found that the PGI_2_ synthase inhibitor tranylcypromine (10 µM) reduced the phenylephrine contraction only in ET-1-incubated segments (Fig. 3a). In agreement, ET-1 increased PGIS gene expression in VSMC from SHR (relative expression 1.46 ± 0.13, *n* = 4; *P* < 0.05). Together, these results suggest that COX-2-derived PGI_2_, via TP receptors, contributes to the ET-1-induced enhancement of phenylephrine-response in MRAs from SHR.

In endothelium-denuded segments, NS398 and SQ29,548 still reduced the phenylephrine contraction in ET-1-incubated segments (Fig. 3b), suggesting that ET-1 increased the production of COX-2-derived vasoconstrictor prostanoids from VSMC.

### Oxidative stress contributes to ET-1-induced COX-2 expression in VSMC from SHR

**Figure 4:**
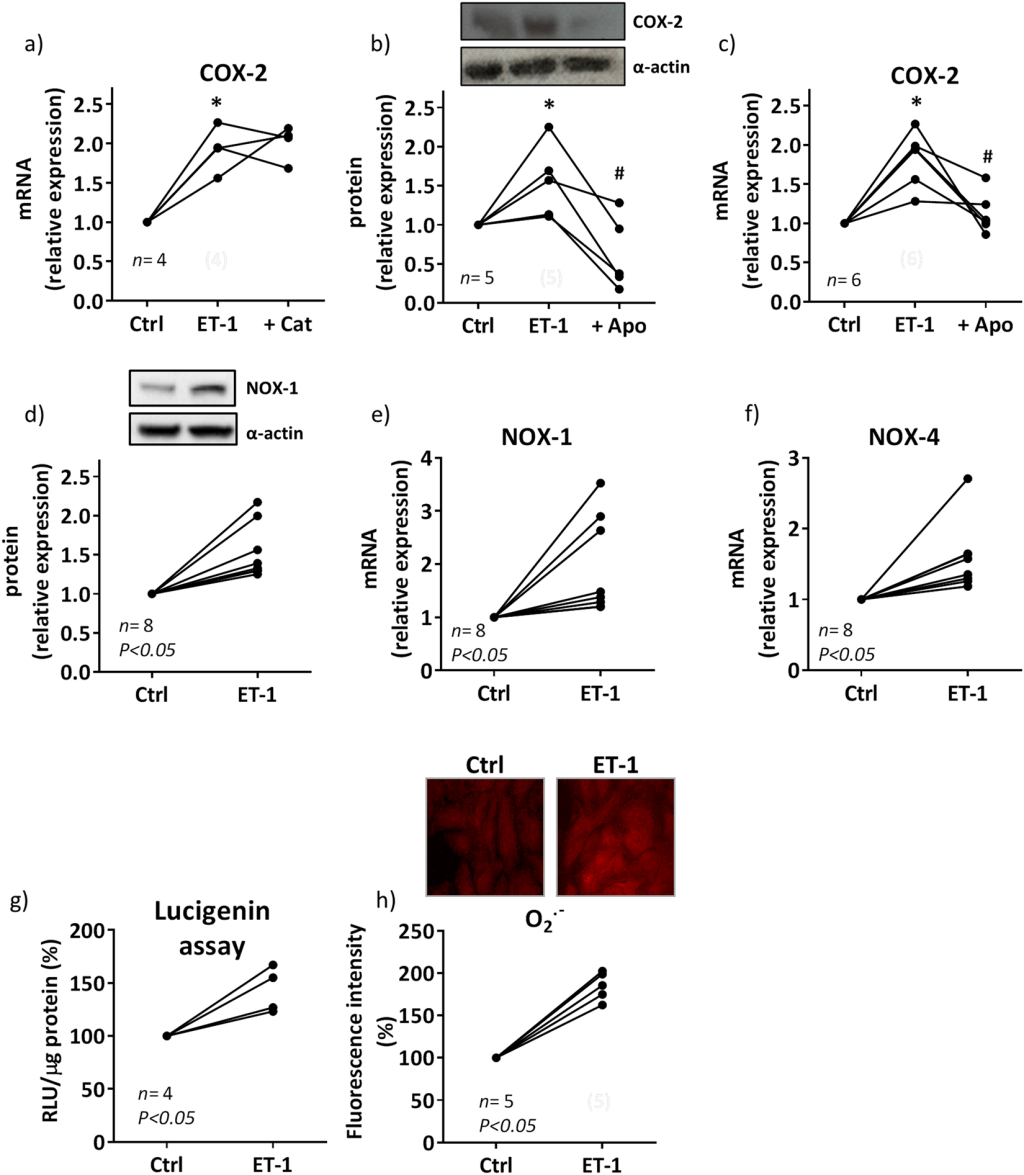
Contribution of oxidative stress to the ET-1-induced COX-2 expression in VSMC from hypertensive rats. (**a**) Effect of catalase (Cat, 1,000 U/ml) on ET-1 (0.1 µM, 1 h)-induced COX-2 mRNA levels. Effect of apocynin (Apo, 30 µM) on ET-1-induced COX-2 protein expression (**b**) and mRNA levels (**c**). Effect of ET-1 on NOX-1 protein expression (**d**) and mRNA levels (**e**), NOX-4 mRNA levels (**f**), NADPH oxidase activity (**g**) and superoxide anion (O_2_^.−^) production (**h**) in VSMC from SHR. Statistical analysis by Student's t-test; *P < 0.05 vs control (Ctrl) and ^#^*P* < 0.05 vs ET-1. *n* denotes number of experiments. Full-length blots are presented in Supplementary Figs S1 and S2.

Next, we analyzed the mechanisms involved in the induction of COX-2 by ET-1 in cultured VSMCs from SHR. As shown in Fig. 4a–c the antioxidant apocynin but not the H_2_O_2_ scavenger catalase (1,000 U/ml), reduced the ET-1-induced COX-2 expression, suggesting the contribution of superoxide anion to the ET-1-induced COX-2 expression. In agreement, ET-1 (0.1 µM) increased NOX-1 protein and mRNA levels (Fig. 4d,e), NOX-4 mRNA levels (Fig. 4f), NADPH oxidase activity and superoxide anion production (Fig. 4g,h).

**Figure 5:**
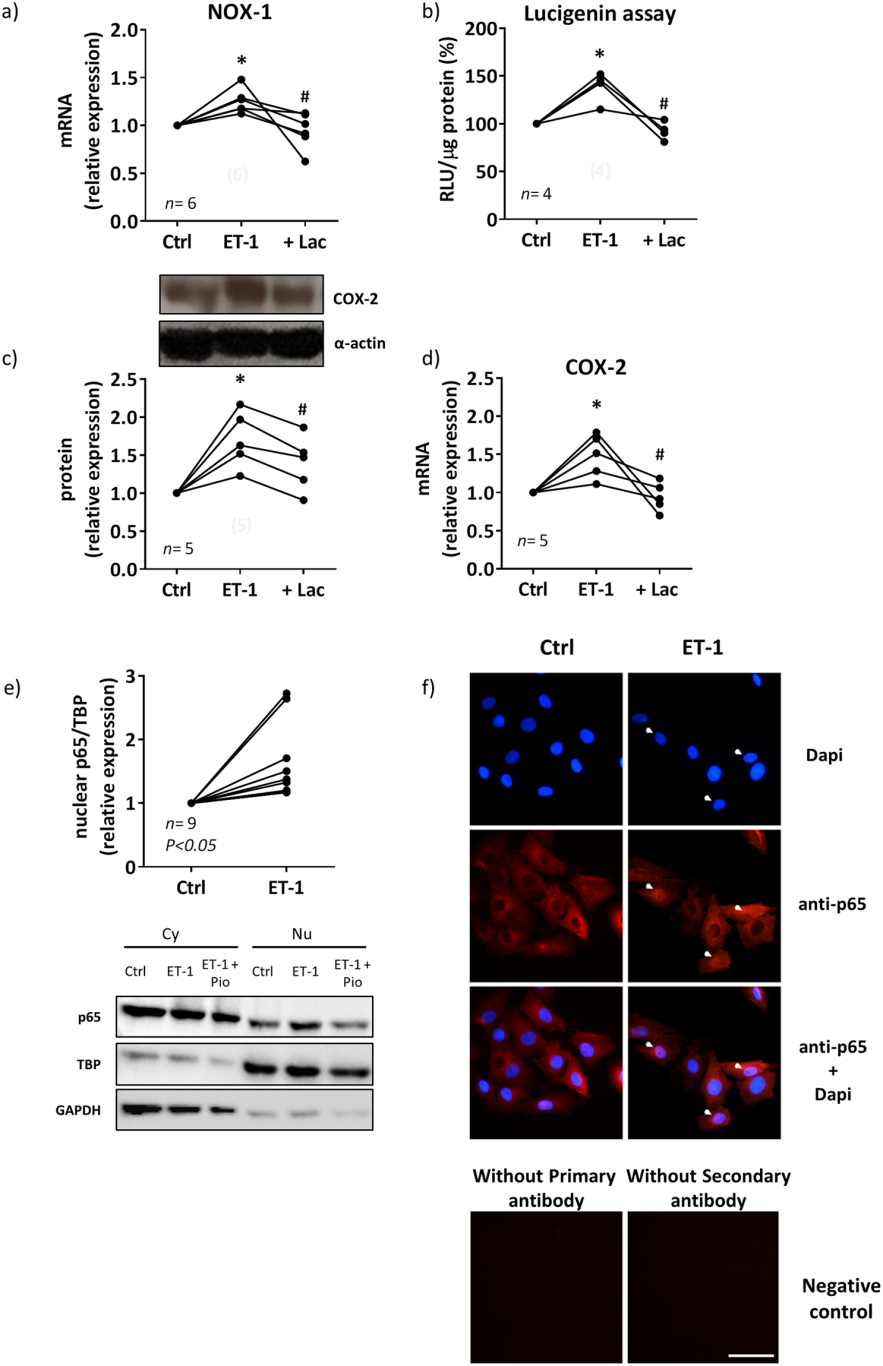
Contribution of NFκB to the ET-1-induced oxidative stress and COX-2 expression in VSMC from hypertensive rats. Effect of lactacystin (Lac, 10 µM) on NOX-1 mRNA levels (**a**), NADPH oxidase activity (**b**), COX-2 protein expression (**c**) and mRNA levels (**d**) induced by ET-1 (0.1 µM, 1 h). (**e**) Effect of ET-1 (0.1 µM, 45 min) on nuclear p65 NFκB protein expression in VSMC from SHR; a representative blot of the cytosolic (Cy) and nuclear (Nu) expression is shown below; TATA-binding protein (TBP) and GAPDH (after reblotting) cytosolic and nuclear expressions are also shown to guarantee the successful cellular fractioning. (**f**) Representative photomicrographs of p65 NFκB immunofluorescence (red) in VSMC from SHR in control and after incubation with ET-1 (0.1 µM, 45 min, *n* = 7). Negative controls without primary or secondary antibody are also shown. Bar scale represents 50 µm. Statistical analysis by Student’s t-test; *P < 0.05 vs control and ^#^*P* < 0.05 vs ET-1. *n* denotes number of experiments. Full-length blots are presented in Supplementary Fig. S3.

It has been described that ET-1 activates several transcription factors^3,34,35^.We then analysed the contribution of nuclear factor kappa-light-chain-enhancer of activated B cells (NFκB) and activator protein 1 (AP-1) on the ROS/COX-2 pathway. In VSMCs from SHR, the proteasome inhibitor lactacystin (10 µM) reduced the ET-1-induced NOX-1 mRNA levels and NADPH oxidase activity (Fig. 5a,b) and COX-2 expression (Fig. 5c,d). In addition, ET-1 increased the nuclear expression of the NFκB p65 subunit (Fig. 5e); the immunofluorescence experiments confirmed the augmented nuclear localization of p65 (Fig. 5f).

**Figure 6:**
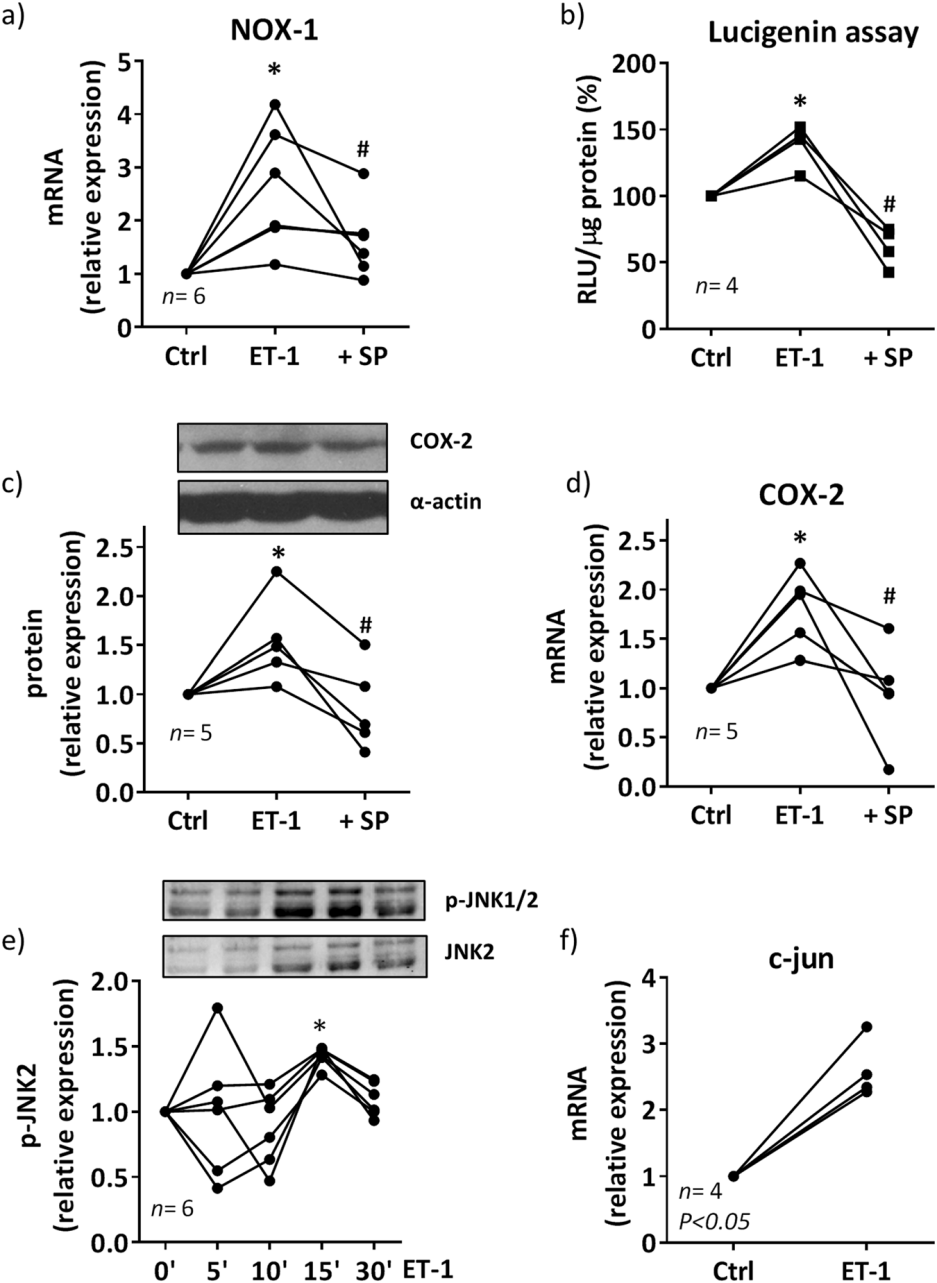
Contribution of AP-1 to the ET-1-induced oxidative stress and COX-2 expression in VSMC from hypertensive rats. Effect of SP600125 (SP, 20 µM) on NOX-1 mRNA levels (**a**), NADPH oxidase activity (**b**) and COX-2 protein expression (**c**) and mRNA levels (**d**) induced by ET-1 (0.1 µM, 1 h). *P < 0.05 vs control (Ctrl) and ^#^*P* < 0.05 vs ET-1 by Student's t-test. (**e**) Time-course of ET-1 (0.1 µM)-induced p-JNK1/2 protein expression in VSMC from SHR; *P < 0.05 vs control (0´) by one-way ANOVA. A representative blot is shown in the upper panel; lower panel shows reblotting of upper panel with JNK2 antibody. (**f**) Effect of ET-1 (0.1 µM, 1 h) on c-jun mRNA levels. Statistical analysis by Student's t-test. *n* denotes number of experiments. Full-length blots are presented in Supplementary Fig. S4.

The specific JNK inhibitor SP600125 (20 µM) also diminished the ET-1-induced NOX-1 expression and NADPH oxidase activity (Fig. 6a,b) as well as COX-2 protein and mRNA levels (Fig. 6c,d). Furthermore, ET-1 increased JNK1/2 phosphorylation and c-jun expression (Fig. 6e,f), supporting the activation of AP-1. Altogether, these results suggest that ET-1 induces COX-2 expression by increasing ROS production through NFκB and AP-1 pathways.

### After pioglitazone treatment of SHR, ET-1 increased NO bioavailability reducing phenylephrine contraction in MRA through ET_B_ receptors

**Figure 7:**
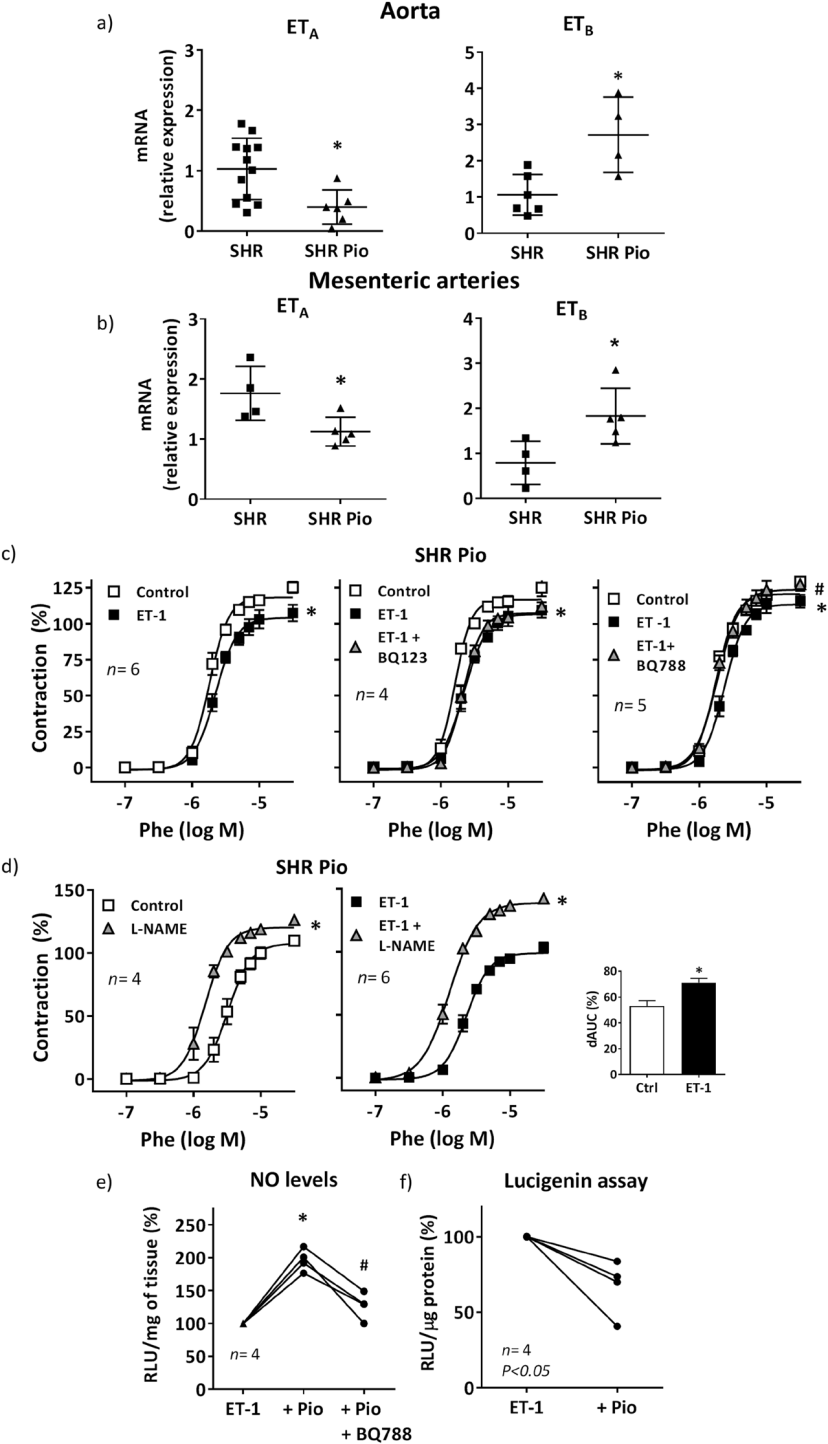
Effect of pioglitazone treatment of hypertensive rats on the vascular ET-1 receptors expression and ET-1 effect on phenylephrine contraction in MRA. Role of nitric oxide. ET_A_ and ET_B_ mRNA levels in aortic (**a**) and mesenteric (**b**) segments from SHR untreated and treated with pioglitazone (Pio, 2.5 mg/Kg·day, 28 days); *P < 0.05 by Student's t test. (**c**) Effect of ET-1 (1 nM, 90 min) and ET-1 plus BQ123 (1 µM) or BQ788 (1 µM) on the concentration-response curve to phenylephrine (Phe) in endothelium-intact segments from pioglitazone (Pio)-treated SHR; *P < 0.05 vs control; ^#^*P* < 0.05 vs ET-1 by two-way ANOVA with repeated measures followed by Sidak or Tukey post hoc test. (**d**) Effect of 0.1 mM L-NAME on the response to Phe in control and ET-1-incubated segments from SHR treated with pioglitazone; *P < 0.05 by two-way ANOVA with repeated measures followed by Sidak post hoc test. Differences of the AUC (dAUC) to Phe in the presence and the absence of L-NAME are also shown; *P < 0.05 by Student's t test. (**e**) Quantification of nitric oxide (NO) levels in vascular segments from SHR incubated with ET-1 (0.1 µM, 1 h) in the absence or the presence of Pio (10 µM, 18 h) and BQ788 (1 µM); *P < 0.05 vs ET-1 and ^#^*P* < 0.05 vs ET-1+Pio by Student's t-test. (**f**) Quantification of NADPH oxidase activity in vascular segments from SHR incubated with ET-1 (0.1 µM, 1 h) in the absence or the presence of Pio (10 µM, 18 h); statistical analysis by Student's t test. *n* denotes number of experiments.

It is well known that glitazones are cardioprotective compounds with antiinflammatory and antioxidant properties^7^. In previous reports, we showed that pioglitazone improved vascular function and reduced the increased vascular ET-1 expression in SHR^5,10^. Herein we found that pioglitazone treatment reduced ET_A_ and increased ET_B_ transcription in aortic and mesenteric segments (Fig. 7a,b). These changes had functional consequences. Thus, in pioglitazone-treated rats, ET-1 reduced phenylephrine contraction, effect prevented by the ET_B_ antagonist BQ788 but not by the ET_A_ antagonist BQ123 (Fig. 7c).

In pioglitazone-treated SHR, L-NAME leftward shifted phenylephrine response in control arteries (Fig. 7d). Notably, the effect of L-NAME was greater in arteries incubated with ET-1 (Fig. 7d), suggesting increased NO availability. Previously we have shown that ET-1 decreased the NO levels; after pioglitazone incubation, the NO levels were increased in ET-1-treated arteries; this increased in NO levels was prevented by BQ788 (Fig. 7e). Additionally, pioglitazone incubation improved the reduction on ACh-induced NO levels observed after ET-1 (NO levels relative to control: ET-1, 76.3 ± 3.2%; ET-1+Pio, 97.0 ± 8.3%, *n* = 5, *P* < 0.05). On the other hand, the NADPH oxidase activity observed in ET-1-treated arteries was reduced by pioglitazone (Fig. 7f). Altogether, these results suggest that after pioglitazone treatment, ET-1/ET_B_ pathway increases NO bioavailability reducing phenylephrine response; the antioxidant properties of pioglitazone likely contribute to this effect.

### Pioglitazone downregulates ET-1-induced COX-2 expression by interference with NFκB and AP-1 activation and reduction of ROS production in VSMC from SHR

**Figure 8:**
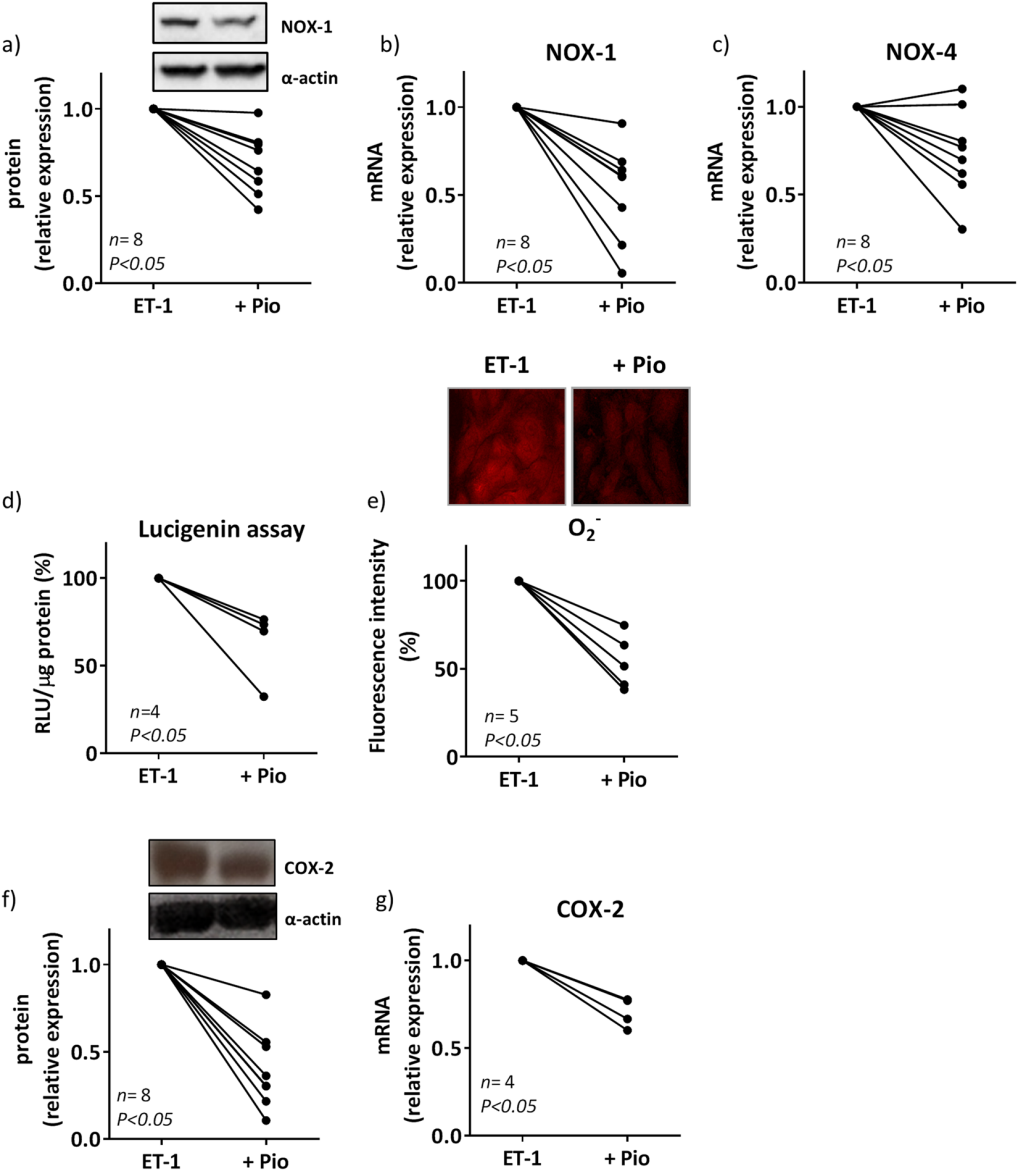
Effect of pioglitazone on the role of oxidative stress to the ET-1-induced COX-2 expression in VSMC from hypertensive rats. Effect of pioglitazone (Pio, 10 µM, 18 h) on ET-1 (0.1 µM, 1 h)-induced NOX-1 protein expression (**a**) and mRNA levels (**b**), NOX-4 mRNA levels (**c**), NADPH oxidase activity (**d)**, superoxide anion (O_2_^−^) production (**e**), and COX-2 protein expression (**f**) and mRNA levels (**g**) in vascular smooth muscle cells from SHR. Statistical analysis by Student's t-test. *n* denotes number of experiments. Full-length blots are presented in Supplementary Figs S2 and S5.

In VSMCs from SHR, pioglitazone prevented ET-1-induced increase of NOX-1 protein and mRNA levels, NOX-4 mRNA levels, NADPH oxidase activity and superoxide anion production (Fig. 8a–e). In addition, pioglitazone reduced ET-1-induced COX-2 mRNA and protein levels (Fig. 8f,g) as well as the mRNA levels of the PGI_2_ synthase (relative expression: 0.71 ± 0.06, *n* = 4, *P* < 0.05).

**Figure 9:**
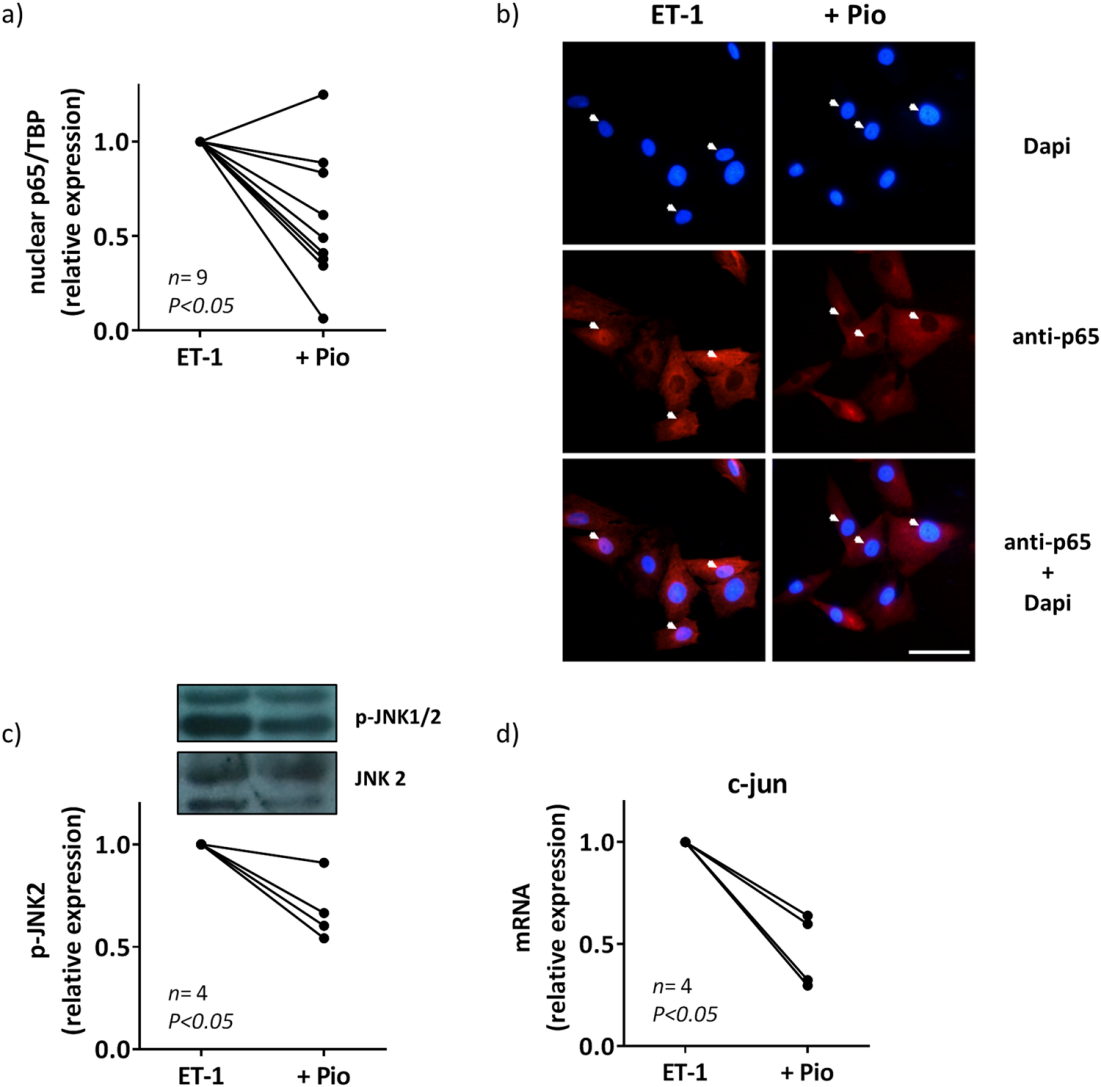
Effect of pioglitazone on the role of NFκB and AP-1 to the ET-1-induced COX-2 expression in VSMC from hypertensive rats. (**a**) Effect of pioglitazone (Pio, 10 µM, 18 h) on ET-1 (0.1 µM, 45 min)-induced nuclear p65 NFκB protein expression in vascular smooth muscle cells (VSMC) from SHR; the representative blot of the cytosolic (Cy) and nuclear (Nu) expression is shown in Fig. 5E. (**b**) Representative photomicrographs of p65 NFκB immunofluorescence (red) in VSMC from SHR after incubation with ET-1 (0.1 µM, 45 min) in the absence and in the presence of pioglitazone. Bar scale represents 50 µm. Effect of pioglitazone on (**c**) p-JNK1/2 protein expression (a representative blot is shown in the upper pane; lower panel shows reblotting of upper panel with JNK2 antibody) and (**d**) c-jun mRNA levels induced by ET-1 (0.1 µM, 15 min for p-JNK2 and 1 h for c-jun). Statistical analysis by Student's t-test. *n* denotes number of experiments. Full-length blots are presented in Supplementary Fig. S6.

Glitazones interfere with NFκB and AP-1 activation by transrepression mechanisms^5,36^. Accordingly, pioglitazone diminished p65 nuclear expression (Fig. 9a,b; representative blot in Fig. 5e), JNK1/2 phosphorylation (Fig. 9c) and c-jun mRNA levels induced by ET-1 (Fig. 9d) in VSMCs from SHR. These results suggest that pioglitazone, by interfering with AP-1 and NFκB, might reduce ROS production and COX-2 expression.

## Discussion

In the present study, we found that ET-1, through ET_A_, increased phenylephrine response in SHR by mechanisms involving production of vasoconstrictor prostanoids from COX-2 and reduction of NO bioavailability, probably because of increased oxidative stress. Pioglitazone treatment of hypertensive animals decreased ET_A_ and increased ET_B_ expression. In these conditions, ET-1 acting on ET_B_, increased NO production and reduced phenylephrine-induced contraction. The antioxidant effect of pioglitazone likely contributed to decrease the COX-2 expression and to increase the NO bioavailability.

ET-1 levels are increased in some hypertension models^5,37^ and this peptide plays a role in hypertension-associated vascular damage^38,39^. ET-1 regulates vascular tone not only by inducing vasoconstriction but also by modulating the response to other vasoactive agents; in addition, the proinflammatory properties of ET-1 seem to contribute to its hypertensinogenic effect^21,22,40^. Here we found greater ET_A_ expression in vessels from SHR than WKY that likely explains the effect of a subthreshold concentration of ET-1 on elevating phenylephrine-induced responses only in SHR. This is in agreement with previous studies showing increased ET_A_ levels in vessels from hypertensive animals^41^ and the involvement of this receptor in the vascular effects of ET-1^40,42,43^; however, the contribution of ET_B_ from VSMC to the vasomotor ET-1 effects in hypertension has also been reported^44^. In this study we did not find alterations in ET_B_ expression or functional effects in hypertension.

L-NAME and endothelium removal increased the phenylephrine contraction in MRA from SHR, being this effect reduced after ET-1 incubation, suggesting that after ET-1, the NO contribution to phenylephrine-induced response is reduced; additionally, ET-1 reduced the basal NO levels, suggesting that, although induces eNOS transcription, ET-1 reduces the NO bioavailability, thus contributing, at least partially, to the increased phenylephrine contraction. In agreement to our results, after NO inhibition, ET-1 failed to further increase the contraction to electrical stimulation and 5-HT, as compared with that obtained in the absence of this peptide^22,42^. Accordingly with that found by other authors^24–26^, after incubation with ET-1 we found increased NADPH oxidase activity; along with this, the NOX inhibitor ML-171 reduced the contraction to phenylephrine in segments from ET-1-treated arteries. All these results suggest that the mechanism responsible for the increased phenylephrine-induced contraction elicited by ET-1 would be the production of ROS from NADPH oxidase reducing NO bioavailability. These would also explain the impairment of ACh-induced relaxation observed after ET-1 incubation. Additional mechanisms include activation of TP receptors by PGI_2_, as shown by the inhibitory effect of the PGI_2_ synthase inhibitor tranylcypromine in phenylephrine-induced contraction in ET-1-incubated segments. Although PGI_2_ is generally associated with vasodilator and antiagregant properties, in conditions such as hypertension or aging PGI_2_ binds TP receptors acting as a vasoconstrictor^10,33^. In addition, this COX-2-derived PGI_2_ is likely produced in VSMC since both NS398 and SQ29,548 reduced the effect of ET-1 on phenylephrine contraction also in endothelium denuded arteries. In agreement, ET-1 induced COX-2 and PGI_2_ synthase expression in VSMC from SHR. The enhanced COX-2 expression was dependent on oxidative stress because the antioxidant apocynin decreased ET-1-induced COX-2 expression and ET-1 increased NOX-1, NOX-4 and NADPH oxidase activity in SHR VSMC. Other authors have described that ET-1 increases ROS production^24–27^ as well as the involvement of oxidative stress in the COX-2 expression induced by several stimuli in different cell types^45^.

The transcription factor NFκB is a downstream element of ET-1 signalling and its involvement in ET-1-induced COX-2 expression has been described in VSMC from SHR-SP^3^. In order to analyze the contribution of NFκB pathway, we used lactacystin, a natural nonpeptidic proteasome inhibitor that prevents degradation of the inhibitory proteins IκBs of the NFκB complex^46^. Lactacystin reduced ET-1-induced NOX-1 expression, NADPH oxidase activity and COX-2 expression in SHR VSMC, supporting the role of NFκB on ET-1/ROS/COX-2 pathway. In agreement, ET-1 increased the nuclear translocation of the p65 NFκB subunit. Moreover, AP-1 seems to play a role in ET-1-induced ROS/COX-2 expression because ET-1 increased JNK1/2 phosphorylation and c-jun mRNA levels in cells from SHR and SP600125 reduced ET-1-induced oxidative stress and COX-2 expression. In agreement, AP-1 dependent modulation of COX-2 was described in endothelial cells from brain mouse^47^, although other authors also showed COX-2 modulation by other kinases in response to ET-1 in microvascular VSMC^48^; therefore, we cannot exclude the contribution of other signalling pathways in the ET-1 effect observed in our conditions.

PPARγ agonists have cardioprotective effects associated, among others, with improvement of vascular inflammation^7^. Additionally, we and others have described that pioglitazone reduces ET-1 production, which might also contribute to its cardioprotective properties^5,31,32,49^. Furthermore, PPARγ regulates ET-1 activated processes such as vasoconstriction^50^ and cardiac hypertrophy^51,52^ and a recent report suggests that specific VSMC PPARγ counteracts ET-1-induced vascular damage^6^. Among the mechanisms responsible for these effects, earlier studies demonstrated that rosiglitazone reduces the contraction to ET-1 by reducing ET_A_ and increasing ET_B_ expression^53,54^, probably due to the fact that ET_B_ gene is a direct target of the transcription factor PPARγ^53^. In agreement, we found lower levels of ET_A_ and higher levels of ET_B_ in vessels from pioglitazone-treated SHR that explains the ET-1/ET_B_-dependent reduction of phenylephrine contraction in these animals. Mechanistically, this effect was due to enhanced NO production as demonstrated by the greater effect of L-NAME on phenylephrine contraction and by the augmented ET_B_-dependent NO release in vessels from pioglitazone-treated SHR.

It has been described that PPARγ agonists reduce oxidative stress^4,5,55,56^. Here we found that pioglitazone treatment reduced the ET-1-induced oxidative stress in SHR vessels and VSMCs; then, we can conclude that the increased NO availability observed after pioglitazone treatment likely results from its antioxidant effects. This might also explain the reduction of ET-1-induced COX-2 expression observed in VSMCs from SHR, which is highly dependent on ROS, as demonstrated in this study. This is consistent with the reduction of COX-2 expression induced by several proinflammatory stimuli after PPARγ activation^3–5^. What is more, pioglitazone also reduced ET-1-induced PGIS levels. Furthermore, pioglitazone attenuated NFκB and AP-1 activation induced by ET-1. In agreement, SMC PPARγ reduces proinflammatory gene expression by inhibition of NFκB activity by promoting p65 nuclear export^57^ but to our knowledge, this is the first time that interference of pioglitazone with ET-1-induced AP-1 activation is described.

## Conclusion

**Figure 10:**
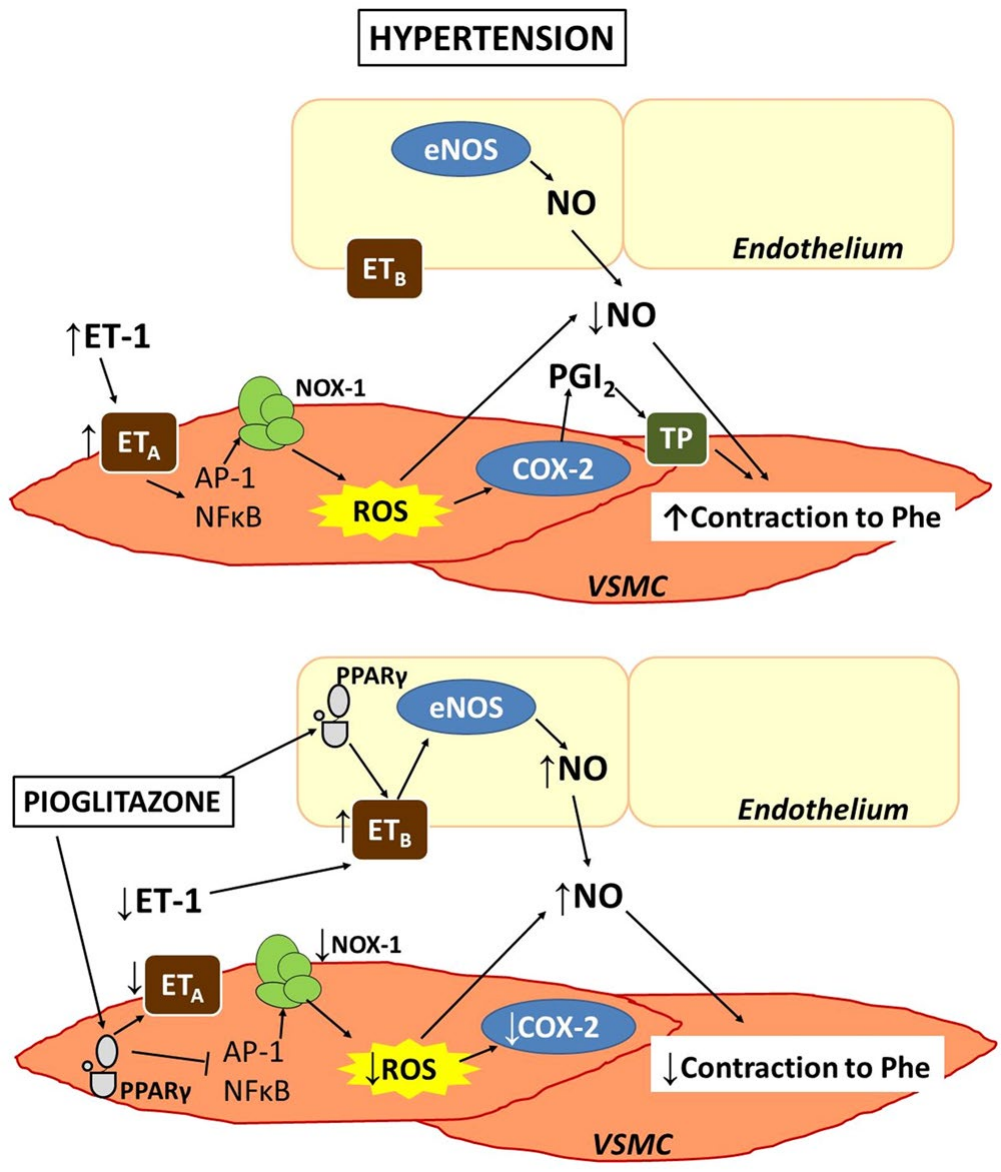
Scheme illustrating the proposed mechanism by which ET-1 increases phenylephrine-induced contraction in hypertension and the effect of PPARγ activation. In segments from hypertensive rats, ET-1, acting on ET_A_ receptors, increases NOX-1 mRNA levels and NADPH oxidase activity through NFκB and AP-1 activation in vascular smooth muscle cells. This enhanced ROS production induces COX-2 expression that together with augmented PGIS expression lead to PGI_2_-dependent activation of TP receptors that increases the contraction induced by phenylephrine; in addition, the increased oxidative stress reduces NO bioavailability, which also contributes to ET-induced effects on phenylephrine response. After pioglitazone treatment, there is a reduction of smooth muscle ET_A_ levels and an increase of endothelial ET_B_. In these conditions, ET-1, acting on ET_B_, increases NO production; the reduction of oxidative stress due to attenuated NFκB and AP-1 activities induced by pioglitazone contributes to decrease COX-2 expression and to increase NO bioavailability; these mechanisms play a role in the reduction induced by ET-1 of the phenylephrine response observed after pioglitazone treatment.

Our results suggest that reduction of NO bioavailability and increased COX-2-derived PGI_2_ production, both mediated by oxidative stress, contribute to the hypercontractility to phenylephrine induced by ET-1 acting on ET_A_ in vessels from hypertensive animals (Fig. 10). Treatment with pioglitazone along with reducing the ET-1 levels previously described^5^, also decreases ET_A_ levels while increases those of ET_B,_; in these conditions, ET-1 through ET_B_ increases the NO production. Additionally, pioglitazone reduces the ET-1-induced oxidative stress, contributing to increase NO bioavailability and to decrease COX-2 expression. All these mechanisms are responsible for the reduction of phenylephrine contraction induced by ET-1 after pioglitazone treatment (Fig. 10). These effects of glitazones on the ET-1 pathway would contribute to improve the cardiovascular alterations observed in hypertension and in other pathological diseases such as diabetes or cardiac hypertrophy^32,49,55,58^. Although ET-1 receptor antagonists have been demonstrated to be useful in the treatment of hypertension, they have not been approved yet in the clinic due to their side effects^59^. Because of the ability of pioglitazone to interfere with ET-1 system as well as to increase peripheral insulin sensitivity, this compound might be a promising therapeutic option in patients with resistant hypertension and in those with hypertension concomitant with insulin resistance.

## Material and Methods

### Animals

All experimental procedures were approved by the Ethical Committee of Research of the Universidad Autónoma de Madrid and Dirección General de Medio Ambiente, Comunidad de Madrid, Spain (PROEX 345/14). Animal care and experimental procedures conformed to the current Spanish laws (RD 53/2013) and are also conformed to the Directive 2010/63/EU of the European Parliament for animal experiments. The studies also comply with the ARRIVE guidelines for reporting experiments involving animals. A total of 15 male WKY (355.7 ± 13.3 g) and 60 SHR (359.4 ± 8.2 g) rats five-six month-old were used.

Animals were obtained from colonies maintained at the Animal Quarters of the Facultad de Medicina of the Universidad Autónoma de Madrid. During treatment, rats were housed with constant room temperature, humidity and light cycle (12-h light/dark) and they had free access to tap water and were fed with standard rat chow ad libitum. The SHR rats were divided into two groups: control (received vehicle) and rats treated with the PPARγ agonist pioglitazone (2.5 mg/Kg·day, for 28 days suspended in 0.5% methylcellulose and administered in drinking water); this dose has been reported to achieve a concentration equivalent to that reported in humans who were administered a 15 mg dose of pioglitazone^60^. Previously we have reported that this treatment did not modify either blood pressure or body weight^10^. At the end of the treatment, rats were killed by decapitation and the mesenteric arcade was removed and placed in Krebs Henseleit solution (KHS) of the following composition (in mM): NaCl 115.0; KCl 4.7; CaCl_2_ 2.5; KH_2_PO_4_ 1.2; MgSO_4_.7H_2_O 1.2; NaHCO_3_ 25.0; glucose 11.1 and Na_2_EDTA 0.01, maintained at 4 °C and continuously gassed with 95% O_2_ and 5% CO_2_. Segments of third-order branches of the mesenteric artery (MRA) were dissected free of fat and connective tissue and used for vascular function studies. Aortas were used to isolate VSMC as well as to analyse the vascular mRNA and NO levels and the NADPH oxidase activity.

### Reactivity experiments

Ring segments, 2 mm in length, were mounted in a small vessel dual chamber myograph for isometric tension measurement^10^. Segments contractility was tested by an initial exposure to a high K^+^ solution (120 mM K^+^-KHS, which was identical to KHS except that NaCl was replaced by KCl on an equimolar basis). The response to K^+^-KHS was similar (*P* >  0.05) in arteries from the three groups (in mN/mm: WKY: 3.04 ± 0.07, *n* = 15; SHR: 3.12 ± 0.09, *n* = 24; SHR pioglitazone: 3.23 ± 0.13, *n* = 13). The presence of endothelium was determined by the ability of 10 μM acetylcholine (ACh) to relax arteries precontracted with phenylephrine at a concentration that produce approximately 50% of the contraction induced by K^+^-KHS in each case. Thereafter, the vascular segments were incubated with 1 nM ET-1 90 min prior to perform a cumulative concentration-response curve to phenylephrine (0.1–30 µM). The effects of indomethacin, NS398, SQ29,548, SC19220, furegrelate, tranylcypromine, L-NAME and ML-171 (2-acetylphenothiazine) were analysed by their addition 30 min before the phenylephrine concentration-response curve. The effects of BQ123 and BQ788 on the ET-1-induced effect were analysed by their addition 30 min before ET-1. In some experiments, endothelium was mechanically removed and the effect of ET-1 on the phenylephrine response was analysed in the absence and the presence of NS398 and SQ29,548. Endothelium removal was assessed by the inability of 10 μM ACh to produce vasodilation. In order to analyze the effect of ET-1 on ACh-induced relaxation, vessels were incubated for 90 min prior to phenylephrine contraction and then a cumulative concentration-response curve to ACh was performed; in some segments, L-NAME was added 30 min before phenylephrine.

Vasoconstrictor responses were expressed as a percentage of the tone generated by K^+^-KHS.

### Cell cultures

Primary cultures of VSMC were obtained from thoracic aortas of 5-month-old SHRs. For this, aortas were aseptically removed, cleaned of fat tissue and blood cells, and placed in DMEM (Invitrogen Life Technologies, Carlsbad, CA, USA) at 4 °C containing 2 mg/ml collagenase type 2 (Invitrogen Life Technologies), 0.1% BSA, 200 U/ml penicillin and 200 μg/ml streptomycin (Sigma Chemical Co., St. Louis, MO, USA). After carefully removal of adventitia, VSMC were obtained by the explant method^5^. Cells from passages 3–8, made quiescent by incubation in DMEM containing 0.2% FBS for 24 h, were used. Cells were identified as VSMCs by the typical spindle shape, by the typical “hills and valleys” distribution, and by positive immunocytochemical staining with specific monoclonal anti-α-actin antibody (Sigma Chemical catalogue number A2547, lot 084M4795V). Cells were stimulated with 0.1 μM ET-1 (for the times indicated in each case) with or without pretreatment for 18 h with pioglitazone or for 1 h with apocynin, catalase, SP600125 or lactacystin.

### Protein expression determination by Western blot

The protein expression was determined by western blot in total extracts (20–25 µg) or nuclear and cytosolic extracts (15 µg) from VSMC. Proteins were separated by 10% SDS-PAGE and transferred to polyvinyl difluoride membranes that were incubated with rabbit polyclonal antibodies for COX-2 (1:250; Cayman Chemical, Ann Arbor, MI, USA, catalogue number 160106, lot 0439169–1), NOX-1 (1:1,000; Abcam, Cambridge, UK, catalogue number ab131088, lot GR3244226-4), p65 (1:1,000, Santa Cruz Biotechnology, Santa Cruz, CA, USA, catalogue number sc-372, lot BO513) or p-JNK1/2 (1:1,000, Cell Signaling Technology, Danvers, MA, USA, catalogue number #9251, lot 24). After being washed, membranes were incubated with anti-rabbit (1:5,000, Bio-Rad, Laboratories, Hercules, CA, USA, catalogue number 70-6515, lot L005679A) or anti-mouse (1:5,000, StressGen, Ann Arbor, MI, USA, catalogue number SAB-100, lot 02020904) IgG antibody conjugated to horseradish peroxidase. The immunocomplexes were detected using an enhanced horseradish peroxidase-luminol chemiluminiscence system (ECL Plus, Amersham Biosciences, GE Healthcare, Little Chalfont, UK) and subjected to autoradiography (Minolta Film, Konica Minolta, Wayne, NJ, USA) or with the ChemiDoc^TM^MP Imaging System (Bio-Rad Laboratories). Signals on the immunoblot were quantified using the Typhoon 9210 quantification software (Amersham Biosciences) or the Image lab Software version 6.0 (Bio-Rad Laboratories). The same membrane was used to determine the expression of α-actin (mouse, 1:250,000, Sigma Chemical Co.) and JNK2 (rabbit, 1:1,000, Cell Signalling Technology, catalogue number #9258, lot 11) in total extract or TATA-binding protein (TBP, rabbit, 1:1,000, Santa Cruz Biotechnology, catalogue number sc-204, lot LO214) and GAPDH (mouse, 1:4,000, Calbiochem, Temecula, CA, USA, catalogue number CB1001, lot 2868983) in nuclear and cytosolic extracts, respectively. Data of protein expression were expressed as the ratio between signals on the immunoblot corresponding to the protein studied and that of α-actin, JNK2 or TBP. To compare the results for protein expression, we assigned a value of 1 to the control or ET-1 situation.

### Immunofluorescence

Proteins were also analysed by immunofluorescence, as previously described^5^. For this, VSMCs were seeded in a 24-well culture plate with coverglasses in the bottom surface. After 60% confluence was reached, cultures were starved in DMEM with 0.1% FBS for 24 h and then stimulated with 0.1 μM ET-1 (45 min) after treatment or not with pioglitazone (10 μM, 18 h). At the end of the treatment, cells were washed and fixed in 4% paraformaldehyde diluted in PBS and maintained in PBS containing 0.2% BSA. Then, cells were incubated overnight at 4 °C with rabbit polyclonal antibody anti-p65 (1:200 dilution). After being washed, red FITC-conjugated goat anti-rabbit antibody (Molecular Probes, Life Technologies, Paisley, UK) was added to the cells at 1:2,000 dilution in the dark. As negative controls we performed the protocol in cells without either primary or secondary antibodies; no staining was observed in both conditions (Fig. 5f). After washing, cells were incubated for 15 min with 4′,6-di-amidino-2-phenylindole (1:10,000, Invitrogen Life Technologies) to stain nuclei, and ProLong Gold antifade mounting reagent (Invitrogen Life Technologies) was added to the microscope slides. Then, fixed and treated cells were placed and sealed in the slides and allowed to completely dry in the dark until the next day. Immunofluorescence-stained cells were observed under a laser scanning confocal microscope (Nikon, C1plus, Nikon Instruments, Melville, NY, USA) and analysed using ImageJ software (http://rsb.info.nih.gov/ij). The fluorescence intensity of cells was measured at four preset areas per sample, and at least three independent experiments were performed.

### mRNA levels determined by qRT-PCR assay

ET_A_, ET_B_, PPARγ and eNOS mRNA levels were determined in mesenteric and/or aortic segments and COX-2, prostacyclin synthase (PGIS), NOX-1, NOX-4 and c-jun mRNA levels in VSMC. Total RNA was obtained by using TRIzol (Invitrogen Life Technologies). A total of 1 μg of DNAse I treated RNA was reverse-transcribed into cDNA using the High Capacity cDNA Archive Kit (Applied Biosystems, Foster City, CA, USA) in a 50 μl reaction. PCR was performed in duplicate for each sample using 0.5 μl of cDNA as template, 1x TaqMan Universal PCR Master Mix (Applied Biosystems) and 20x of Taqman Gene Expression Assays (Applied Biosystems, COX-2: Rn00568225_m1; NOX-1: Rn00586652_m1; c-jun: Rn00440945_m1) or specific primers (Sigma-Aldrich, ET_A_ Fw: GCCATTGAAATTGTCTCCATCTGG, Rv: GAACTTGGTCGTGGCGTTGA; ET_B_ Fw: GATACGACAACTTCCGCTCCA, Rv: GTCCACGATGAGGACAATGAG; PPARγ Fw: TCGCTGATGCACTGCCTATG, Rv; GGAGTGGTCATCCATCACAG; eNOS Fw: GAGAGTGAGCTGGTGTTTGG, Rv: GGTGAACATTTCCTGTGCTGT; NOX-4 Fw: GCCTCCATCAAGCCAAGA, Rv: CCAGTCATCCAGTAGAGTG; PGIS Fw: CCATCAACAGCATCAAACAGTTT, Rv: CAAAGCCATATCTGCTAAGGTCAA), and Fast Start Universal SYBR Green Master (Rox) in a 20 μl reaction. For quantification, quantitative RT-PCR was carried out in an ABI PRISM 7000 Sequence Detection System (Applied Biosystems, from the CAT of Universidad Rey Juan Carlos) using the following conditions: 2 min 50 °C, 10 min 95 °C and 40 cycles: 15 s 95 °C, 1 min 60 °C. As a normalizing internal control we amplified β2 microglobulin (Rn00560865_m1) and cyclophilin D (Rn01458750_g1). To calculate the relative index of gene expression, we used the 2^−ΔΔCt^ method; for comparisons, we used WKY or untreated SHR as a control situation in vascular segments and control or ET-1-treated cultures in VSMC.

### *In situ* detection of vascular O_2_∙^−^ production

The oxidative fluorescent dye dihydroethidium (DHE) was used to evaluate *in situ* O_2_∙^−^ production in VSMCs, as previously described^5^. Hydroethidine freely permeates cells and is oxidized in the presence of O_2_∙^−^ to ethidium bromide, which is trapped by intercalation with DNA. Ethidium bromide is excited at 546 nm and has an emission spectrum at 600–700 nm. VSMCs were plated onto glass coverslips inserted into six-well plates and cultured as described above. Subconfluent cells were stimulated with ET-1 (0.1 μM) for 1 h in the absence and presence of pioglitazone or apocynin that were respectively added 18 h or 1 h before ET-1. Afterwards, cells were loaded with DHE (10 µM) in serum-free DMEM with 0.1% BSA for 30 min at 37 °C. Nonstimulated VSMCs were imaged daily in parallel using the same image settings. Images were captured with a fluorescent laser scanning confocal microscope (Leica TCS SP2). The fluorescence intensity values of 10–20 nuclei per experiment were measured using the Metamorph Image Analysis Software (Molecular Devices, Downingtown, PA, USA).

### Lucigenin assay

A lucigenin-enhanced chemiluminescence assay was used to determine NADPH oxidase activity in aortic segments and VSMCs. Arterial segments from the same animal and VSMCs from the same batch were incubated or not with ET-1, previously treated or not with pioglitazone, lactacystin or SP600125. Aortic segments or VSMCs were homogenized in lysis buffer (in mM: 50 KH_2_PO_4_, 1 EGTA and 150 sucrose, pH 7.4) on ice to avoid degradation. Protein concentration was determined by using the Bradford reactive and 50 μl of the homogenate (containing 50 µg protein in VSMC and 75–100 µg protein in aortic segments) were transferred to a 96 well plate together with 175 μl of assay phosphate buffer and lucigenin (5 µM); the assay was performed by duplicate. Basal luminescence was measured every 1.8 seconds during 3 minutes in a plate luminometer (Auto-Lumat LB 953, Berthold Technologies, Bad Wildbad, Germany). The reaction was started by the addition of NADPH (0.1 mM) and luminescence was measure every 1.8 seconds during 3 minutes. The buffer blank was subtracted from each reading. Activity was expressed as relative light units per μg of protein. Variations of luminescence were calculated as the amount relative to control or ET-1-treated arterial segments from the same animal or VSMCs from the same batch.

### NO release

NO levels were analyzed in aortic segments from SHR rats with the fluorescent probe 4,5-diaminofluorescein (DAF-2), as described^29^; each day, one segment was used as control and others from the same animal were incubated with ET-1 previously treated or not with pioglitazone with or without BQ788. Then, vascular segments were stabilized in a Krebs-HEPES buffer (in mM: 119 NaCl; 20 HEPES; 1.2 CaCl_2_; 4.6 KCl; 1 MgSO_4_; 0.4 KH_2_PO_4_; 5 NaHCO_3_; 5.5 glucose; 0.15 Na_2_HPO_4_, pH 7.4) at 37 °C for 30 min. Subsequently, segments were incubated with DAF-2 (20 µM in Krebs-HEPES) at 37 °C for 45 min. Segments were made permeable with 0.05% triton for 3 min, and the levels of NO were measured using a fluorometer (FLUOstar OPTIMA, BGM Labtech, Ortenberg, Germany). To analyze the effect of ET-1 on ACh-induced NO release, some segments were incubated with phenylephrine (1 µM, 5 min) and ACh (10 µM, 10 min). Thereafter, the medium was collected to measure agonist-induced NO release. The induced NO release was calculated by subtracting basal NO release from that evoked by ACh. At the end of the experiments, aortic segments were weighted to correct the NO release. For negative controls, one segment was incubated as above indicated with 0.1 mM L-NAME. A tube without aortic segment was included as blank. The amount of NO released was expressed as arbitrary units per mg of dry tissue. Variations of NO release were calculated as the amount relative to control or ET-1-treated segments from the same animal.

### Data analysis

All values are expressed as mean ± standard error (S.E.M.); *n* denotes the number of animals or the number of different cultures (each one obtained from three different animals) used in each experiment. In vascular reactivity experiments, the maximum response (Emax values) and the negative logarithm of concentrations of phenylephrine producing 50% of maximum response (pD_2_ values), were calculated by a non-linear regression analysis of each individual concentration-response curve using a computer program (GraphPad Prism Software, San Diego, CA, USA). To compare the effects of L-NAME or endothelium removal on the response to phenylephrine in segments from different groups, results are also expressed as differences of area under the concentration-response curve (dAUC) in control and experimental situations. AUCs were calculated from the individual concentration-response curve plots using the GraphPad Prism Software; the differences were expressed as a percentage of the AUC of the corresponding control situation. In the cell culture experiments, since the different wells of one plate have been seeded with the same batch of cells, we consider the other wells as the same subject; therefore, data are expressed as a n-fold increase relative to control in each plate, value of which was 1. Results were analysed by using Student's t-test or two-way ANOVA with or without repeated measures followed by Tukey or Sidak test for multiple comparisons by using the GraphPad Prism Software version 7.04. *P* ≤0.05 was considered as significant differences.

### Materials

Phenylephrine, ACh, SC19220, furegrelate, tranylcypromine, L-NAME, ML-171, catalase, lactacystin, SP600125, indomethacin, BQ123 and BQ788 were obtained from Sigma Chemical, Co. NS398 was obtained from Calbiochem-Novabiochem GmbH (Bad Soden, Germany), apocynin from Fluka-Sigma Chemical (Seelze, Germany) and SQ29,548 from Cayman Chemical. Pioglitazone was generously supplied by Takeda-Lilly, Madrid, Spain.

## Supplementary information

Supplementary information

## Data Availability

All data generated or analyzed during this study are included in this article (and its Supplementary Information file).
