# Supplementary material for: Pioglitazone Modulates the Vascular Contractility in Hypertension by Interference with ET-1 Pathway
Source: Sci Rep. 2019 Nov 11;9:16461. doi: 10.1038/s41598-019-52839-6 (PMC6848177; doi:10.1038/s41598-019-52839-6)

Pioglitazone Modulates the Vascular Contractility in Hypertension by Interference with ET-1 Pathway

Roberto Palacios-Ramírez[1,2,+], Raquel Hernanz[1,2,+], Angela Martín[1,2], José V. Pérez-Girón[1,#], María T. Barrús[1,2], Zoe González-Carnicero[1], Andrea Aguado[3], Frederic Jaisser[4], Ana M. Briones[2,3], Mercedes Salaices[2,3], María J. Alonso[1,2,*]

Manuscript number SREP-18-48860A

Raw Data Figures

Fig. S1. Original uncropped images of blot shown in Figure 4 Bands in squares correspond to those shown in the corresponding Figure 4b

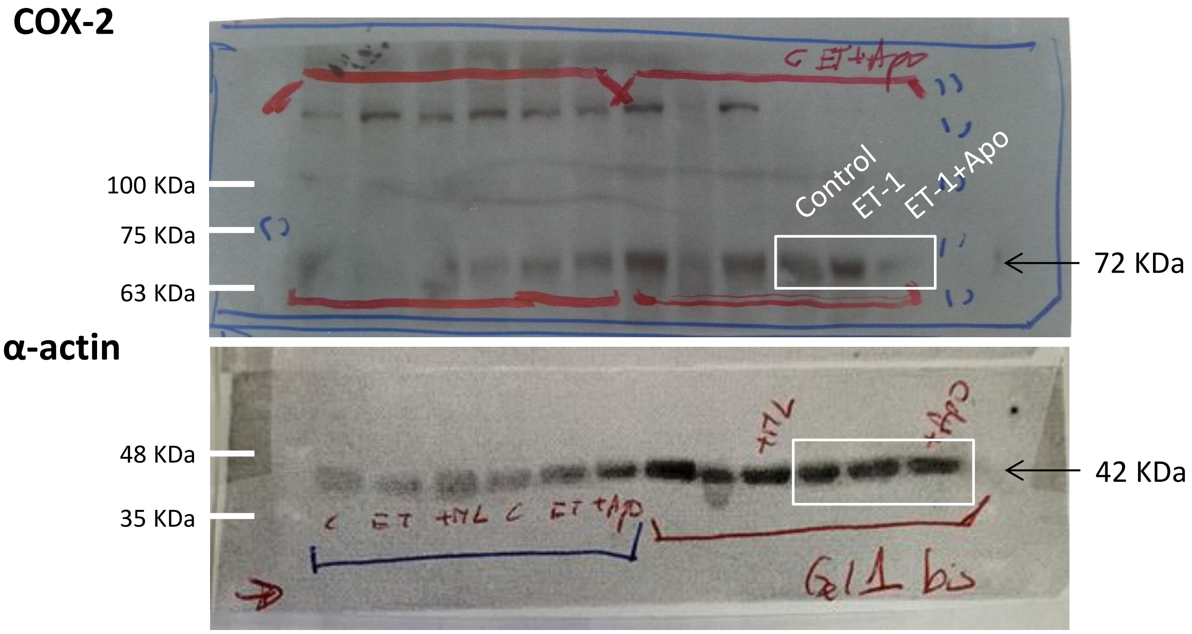

Fig. S2. Original uncropped images of blot shown in Figures 4 and 8. Bands in squares correspond to those shown in the corresponding Figures 4d and 8a.

NOX-1

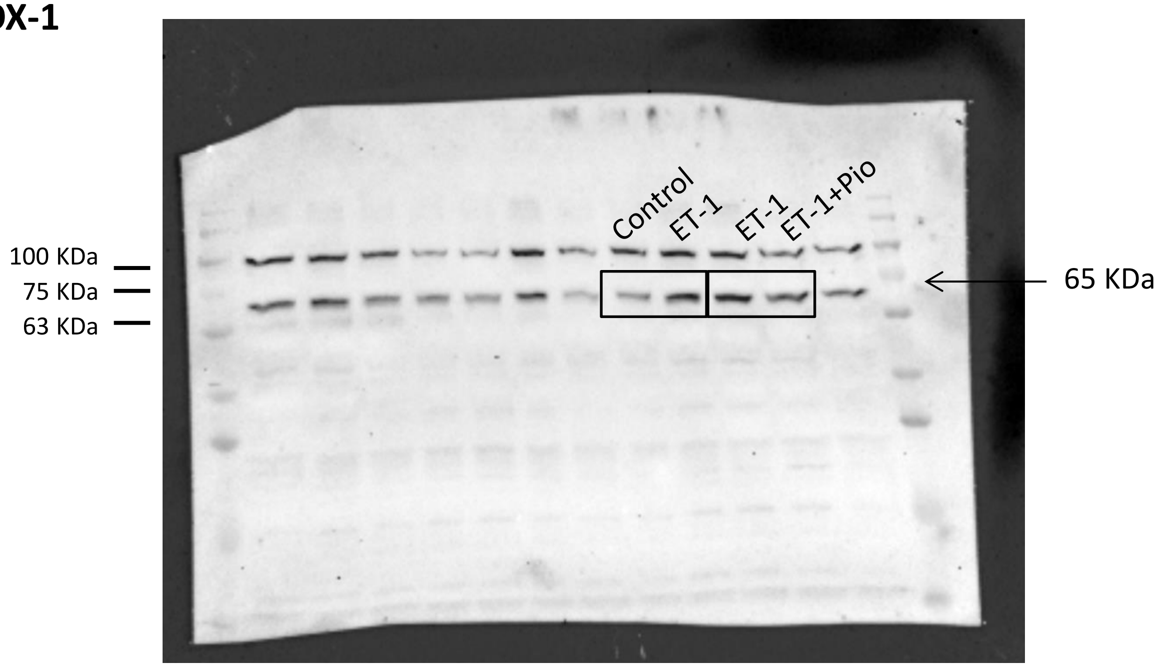

α-actin

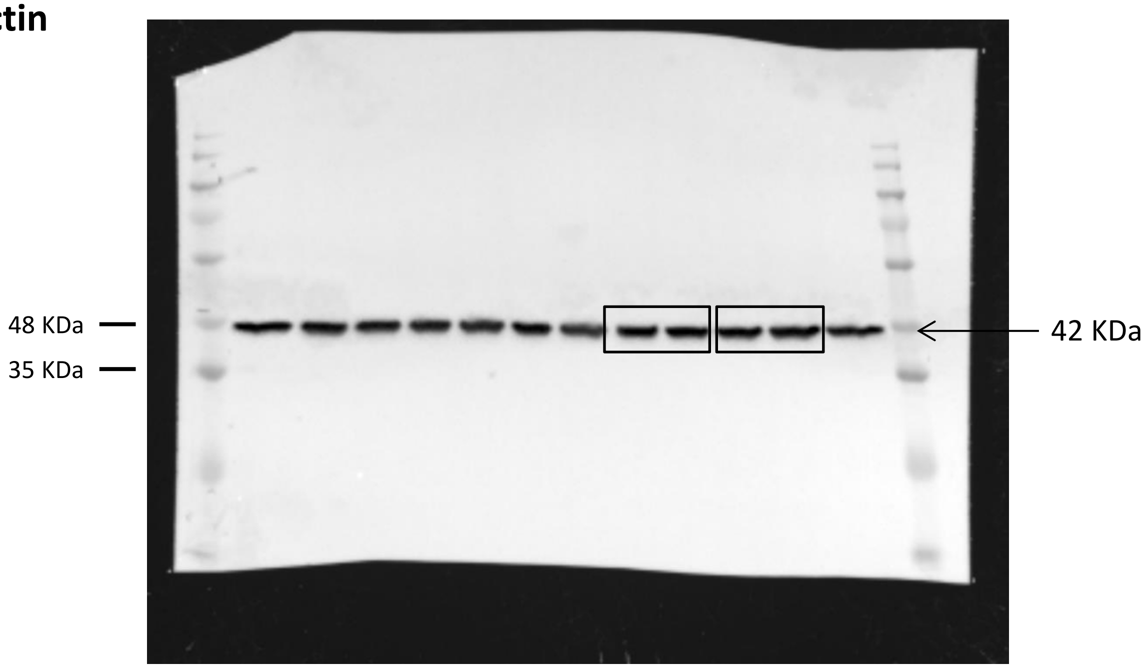

Fig. S3. Original uncropped images of blot shown in Figure 5. Bands in squares correspond to those shown in the Figure 5c (a) and Figure 5e (b).

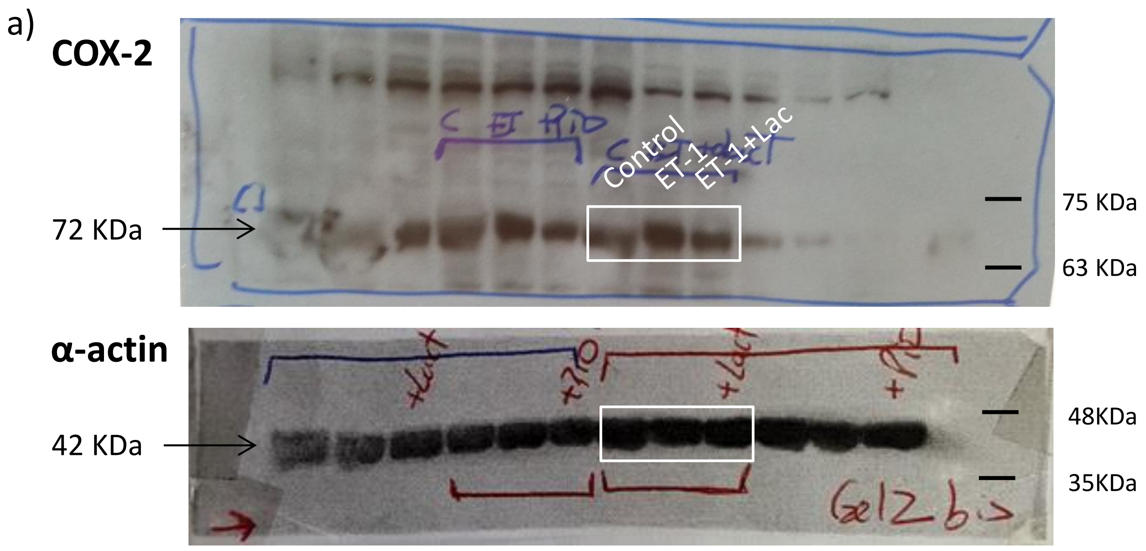

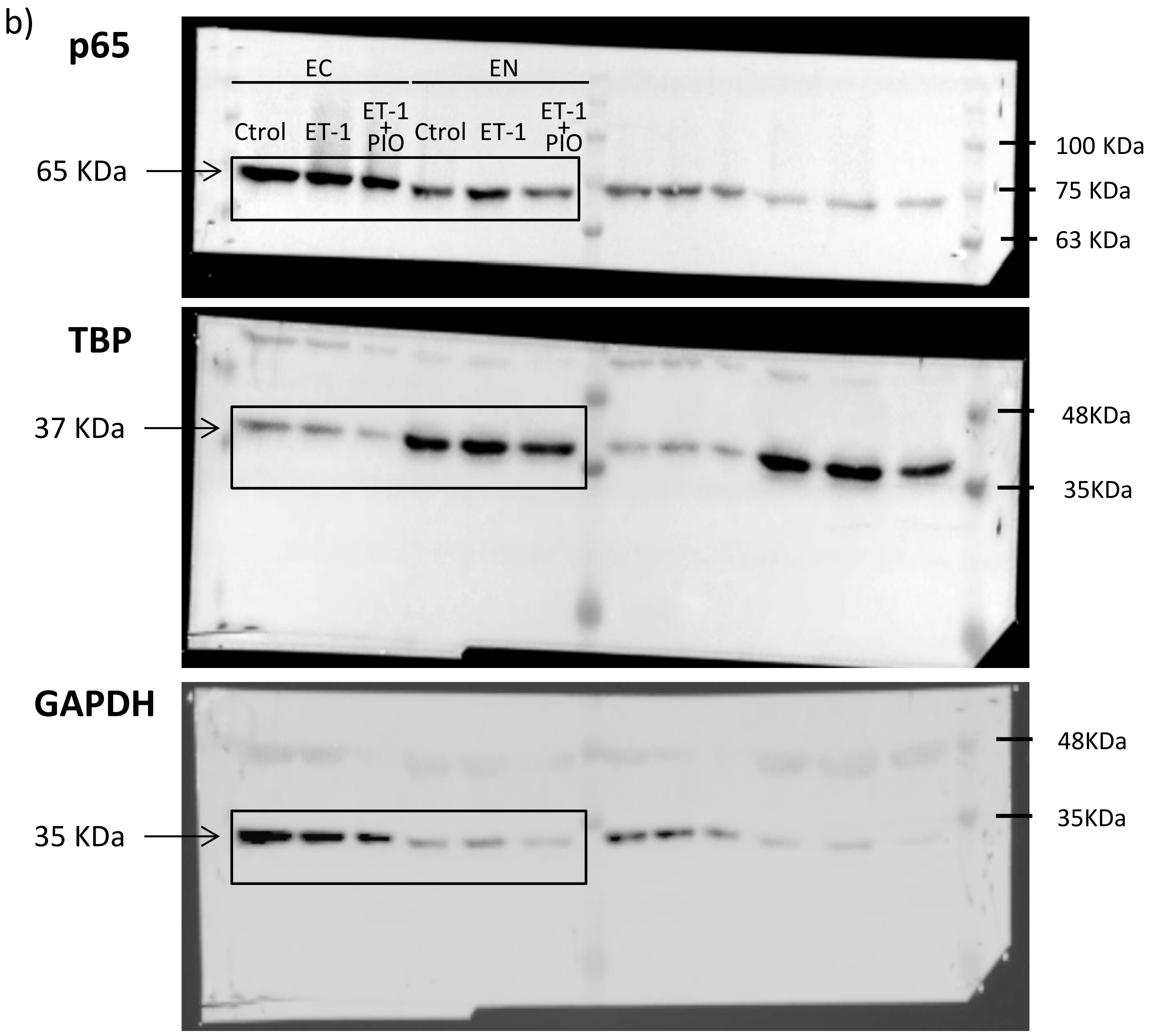

Fig. S4. Original uncropped images of blot shown in Figure 6. Bands in squares correspond to those shown in the Figure 6c (a) and Figure 6e (b).

a)

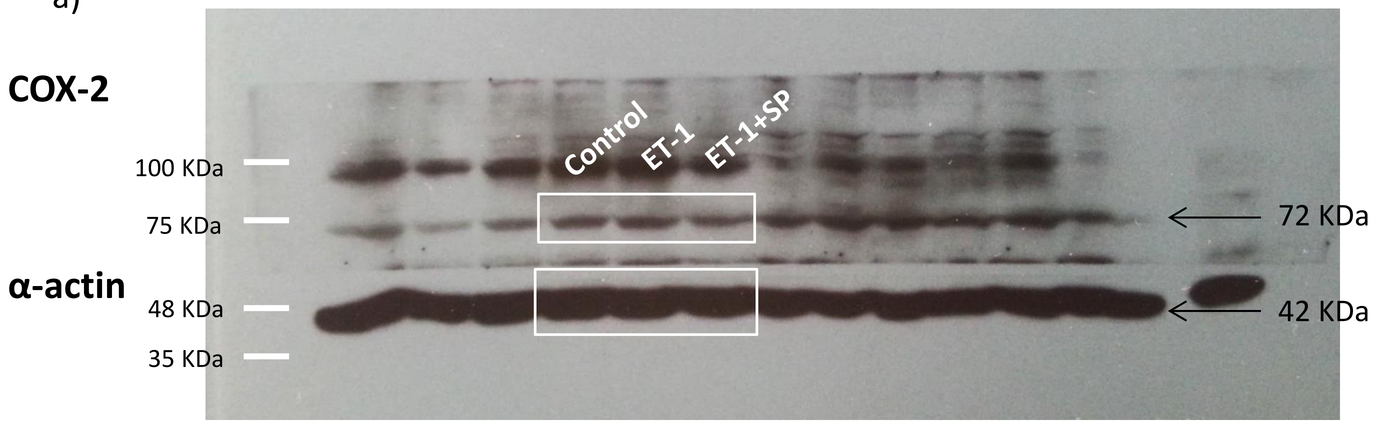

b)

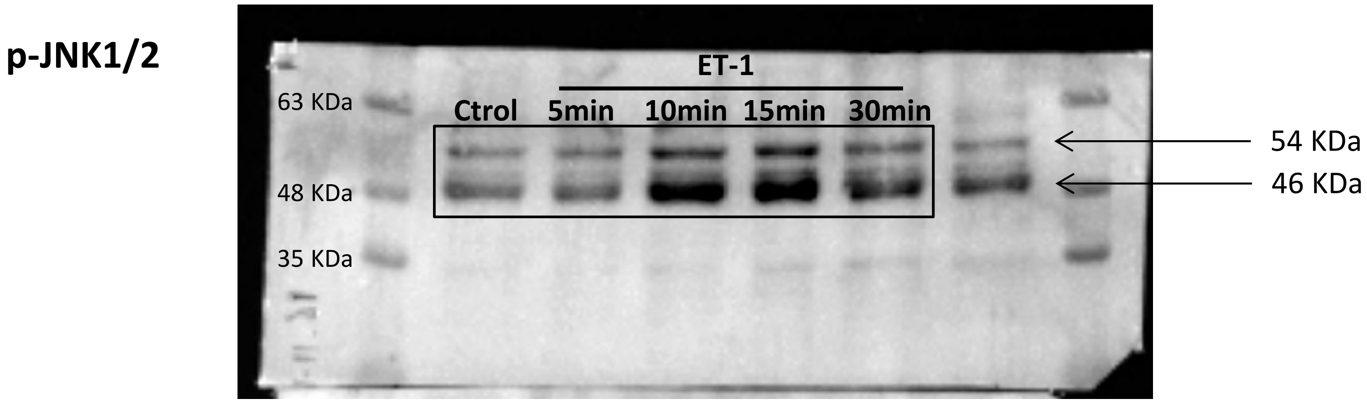

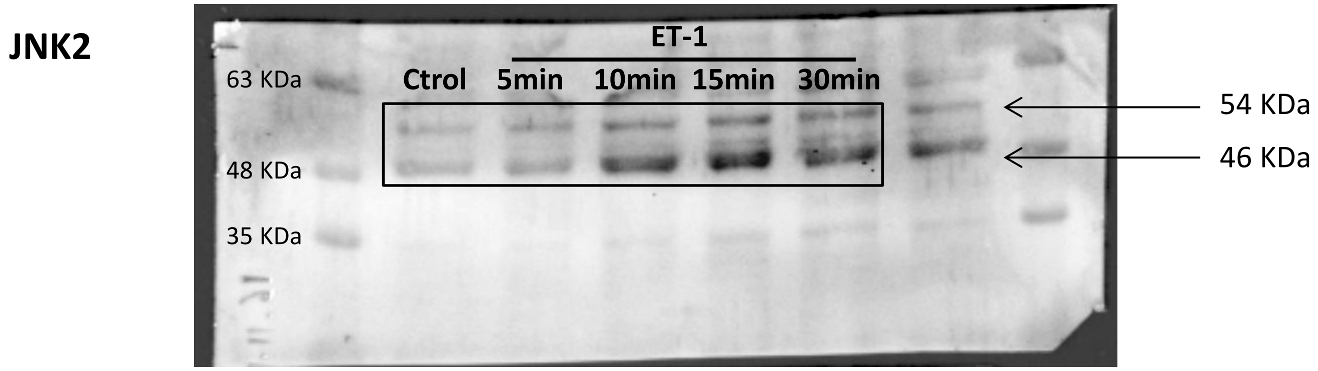

Fig. S5. Original uncropped images of blot shown in Figure 8. Bands in squares correspond to those shown in the corresponding Figure.

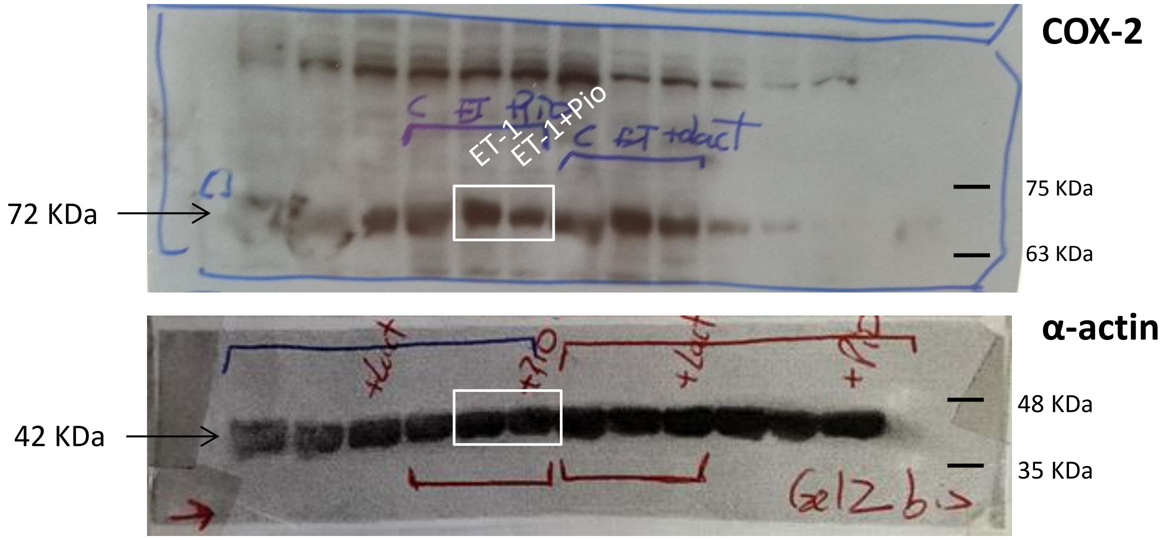

Fig. S6. Original uncropped images of blot shown in Figure 9. Bands in squares correspond to those shown in the corresponding Figure.

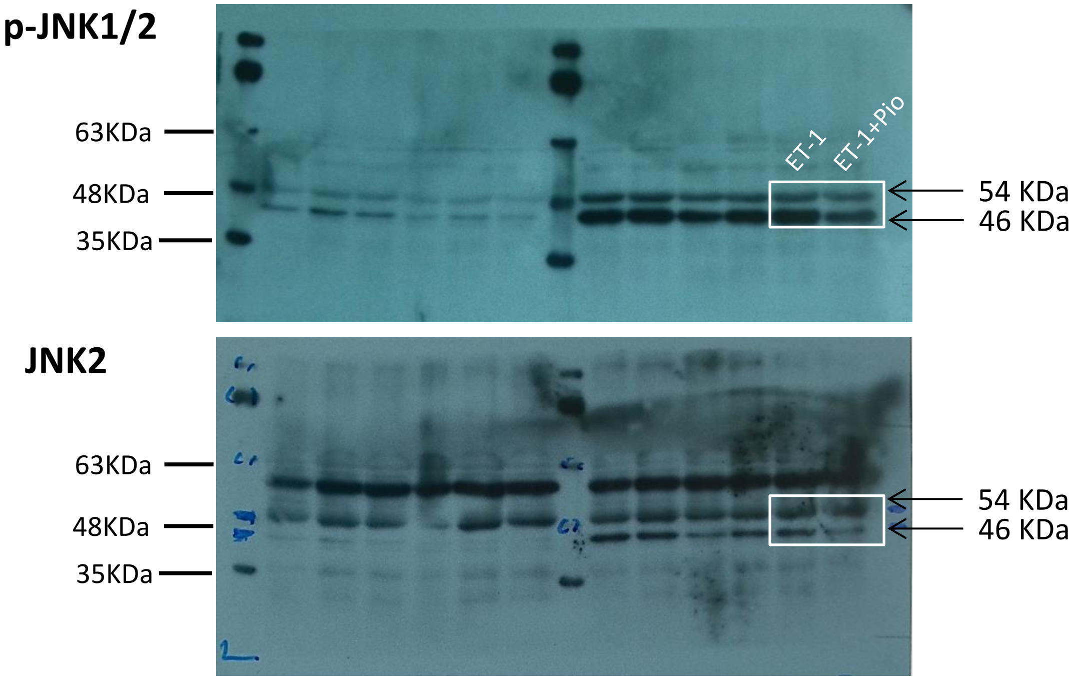

Supplement: Supplementary file 1 — Supplementary information [file 41598_2019_52839_MOESM1_ESM.pdf]
